# Nucleoporin POM121 signals TFEB-mediated autophagy via activation of SIGMAR1/sigma-1 receptor chaperone by pridopidine

**DOI:** 10.1080/15548627.2022.2063003

**Published:** 2022-05-04

**Authors:** Shao-Ming Wang, Hsiang-En Wu, Yuko Yasui, Michal Geva, Michael Hayden, Tangui Maurice, Mauro Cozzolino, Tsung-Ping Su

**Affiliations:** a Cellular Pathobiology Section, Integrative Neuroscience Research Branch, Intramural Research Program, National Institute on Drug Abuse, National Institutes of Health, DHHS, 333 Cassell Drive, Baltimore, Maryland 21224, USA; b China Medical University, Graduate Institute of Biomedical Sciences, Taiwan; c Neuroscience and Brain Disease Center, China Medical University, No.91, Hsueh-Shih Road, Taichung city, 404333, Taiwan; d Department of Neurology, China Medical University Hospital, No.2, Yude Road, North District, Taichung city, 404333, Taiwan; e Prilenia Therapeutics Development Ltd, Herzliya, Israel; f The Centre for Molecular Medicine and Therapeutics, BC Children’s Hospital Research Institute, University of British Columbia, Vancouver, British Columbia, Canada; g MMDN, University of Montpellier, EPHE, INSERM, Montpellier, France; h Institute of Translational Pharmacology, CNR, Via del Fosso del Cavaliere 100, 00133, Rome, Italy

**Keywords:** ALS/FTD, c9orf72, chaperone, KPNB1/importinβ1, nucleocytoplasmic transport, nucleoporin POM121, pridopidine, SIGMAR1, sigma-1 receptor, TFEB

## Abstract

Macroautophagy/autophagy is an essential process for cellular survival and is implicated in many diseases. A critical step in autophagy is the transport of the transcription factor TFEB from the cytosol into the nucleus, through the nuclear pore (NP) by KPNB1/importinβ1. In the C9orf72 subtype of amyotrophic lateral sclerosis-frontotemporal lobar degeneration (ALS-FTD), the hexanucleotide (G4C2)RNA expansion (HRE) disrupts the nucleocytoplasmic transport of TFEB, compromising autophagy. Here we show that a molecular chaperone, the SIGMAR1/Sigma-1 receptor (sigma non-opioid intracellular receptor 1), facilitates TFEB transport into the nucleus by chaperoning the NP protein (i.e., nucleoporin) POM121 which recruits KPNB1. In NSC34 cells, HRE reduces TFEB transport by interfering with the association between SIGMAR1 and POM121, resulting in reduced nuclear levels of TFEB, KPNB1, and the autophagy marker LC3-II. Overexpression of SIGMAR1 or POM121, or treatment with the highly selective and potent SIGMAR1 agonist pridopidine, currently in phase 2/3 clinical trials for ALS and Huntington disease, rescues all of these deficits. Our results implicate nucleoporin POM121 not merely as a structural nucleoporin, but also as a chaperone-operated signaling molecule enabling TFEB-mediated autophagy. Our data suggest the use of SIGMAR1 agonists, such as pridopidine, for therapeutic development of diseases in which autophagy is impaired.

**Abbreviations**: ALS-FTD, amyotrophic lateral sclerosis-frontotemporal dementiaC9ALS-FTD, C9orf72 subtype of amyotrophic lateral sclerosis-frontotemporal dementiaCS, citrate synthaseER, endoplasmic reticulumGSS, glutathione synthetaseHRE, hexanucleotide repeat expansionHSPA5/BiP, heat shock protein 5LAMP1, lysosomal-associated membrane protein 1MAM, mitochondria-associated endoplasmic reticulum membraneMAP1LC3/LC3, microtubule-associated protein 1 light chain 3NP, nuclear poreNSC34, mouse motor neuron-like hybrid cell lineNUPs, nucleoporinsPOM121, nuclear pore membrane protein 121SIGMAR1/Sigma-1R, sigma non-opioid intracellular receptor 1TFEB, transcription factor EBTMEM97/Sigma-2R, transmembrane protein 97

## Introduction

Amyotrophic lateral sclerosis-frontotemporal dementia (ALS-FTD) is a devastating neurodegenerative disease for which there is currently no effective treatment. Approximately 40% of familial ALS-FTD are caused by an expanded hexanucleotide (G4C2)RNA repeats (HRE) in the *C9orf72* gene [1,2]. The HRE binds and inhibits the GTPase activating protein RANGAP1 at the nuclear pore (NP), thereby preventing RANGAP1 from activating the Ras-related protein RAN. RAN activation provides energy for the nucleocytoplasmic transport by KPNB1/importinβ1 [3–7].

The NP is a large complex assembly composed of about 30 nuclear pore proteins called nucleoporins (NUPs) [8–10]. The stability and regulation of these NUPs play critical roles in the nucleocytoplasmic transport that facilitates the communication and signaling between the nucleus and extra-nuclear domains in a cell or neuron. The NP is recognized as the gateway to neurodegeneration [11]. Some of the NUPs have short life spans while most have long half-lives [12,13]. Of note, the NUP POM121 plays a gate-keeping role whose stability affects the level of several other NUPs [13]. POM121 is located at the central pore of the NP [13] and is known to bind KPNB1/importinβ1 to facilitate the nucleocytoplasmic transport of transcription factors including E2F1 (E2F transcription factor 1), MYC, and AR (androgen receptor)-GATA2 transcription factor [14]. Despite its importance, little is known about the molecular regulation and downstream effects of POM121 activity.

SIGMAR1/Sigma-1 receptor (sigma non-opioid intracellular receptor 1) [15–27] is a molecular chaperone that resides mainly at the mitochondria-endoplasmic reticulum interface called the MAM, where it chaperones the ITPR3/IP3 receptor type 3 to ensure proper Ca^2+^ signaling from the endoplasmic reticulum into mitochondria for adenosine triphosphate production [15,26,28–30]. SIGMAR1 proteins can also be found in other parts of a cell including the reticular network of endoplasmic reticulum, plasma membrane, nuclear envelope, and NP [15,29,31,32]. Activation of SIGMAR1 exerts neuroprotective functions demonstrated in numerous models of neurodegenerative diseases, including ALS-FTD [33–36]. Importantly, we have shown that the SIGMAR1 at the NP attenuates the deleterious effect of HRE by chaperoning the stability of other NUPs like NUP50, NUP214, and RANBP2/NUP358 [32]. Furthermore, SIGMAR1 proteins serve as a molecular sponge for the (G4C2)RNA repeats at the NP [32]. POM121 plays a key role maintaining the stability of the nuclear pore complex. Therefore, when G4C2 repeat RNA initiates a reduction of POM121 expression in C9orf72 neurons, the G4C2 repeat may lead to decreased expression of several other NUPs [13]. We therefore hypothesized that SIGMAR1 proteins directly chaperone POM121 which leads to the observed beneficial effects of other NUPs. Because POM121 is also a NUP at the NP, the possibility exists that the SIGMAR1 may also chaperone POM121 and regulate its function thereof.

A feature common to all SIGMAR1 agonists is the biphasic dose response, which was observed in numerous *in vitro* and *in vivo* preclinical studies as well as in clinical trials [36–42]. The exact mechanisms underlying the observed biphasic dose response of SIGMAR1 agonists remain unknown. A number of potential mechanisms driving the biphasic response have been proposed, including selectivity for SIGMAR1 vs TMEM97/Sigma-2 R (activation of TMEM97 may counteract SIGMAR1 neuroprotective effects [43,44]) and stabilization of the active SIGMAR1 monomers by the optimal dose [36], while higher doses stabilize SIGMAR1 proteins in a nonactive oligomeric form [45–47] among others [36]. Of note, SIGMAR1 proteins have been shown to exist in oligomeric forms in the presence of antagonists [46,47]. On the contrary, SIGMAR1 agonists reduce the formation of oligomers as shown in several elegant studies [27,46–48].

Pridopidine (4-[3-{methylsulfonyl}phenyl]-1-propylpiperidine) is a highly selective and potent SIGMAR1 agonist (Ki = 0.057 µM) [49]. Pridopidine demonstrates neuroprotective effects, which are exquisitely mediated by activation of the SIGMAR1 in models of several neurological diseases including ALS, Huntington disease, Alzheimer disease and Parkinson disease [50–57]. Interestingly, these effects adhere to the classic biphasic dose response curve [41,58].

Impairments in macroautophagy/autophagy [59] play a critical role in neurodegenerative diseases [60]. (G4C2)RNA repeats were recently shown to cause pathological cytosolic accumulation of TFEB, a key transcriptional regulator of autolysosomal function [61–65]. TFEB is transported from the cytosol into the nucleus by KPNB1/importinβ1 [66]. However, the molecular mechanism by which (G4C2)RNA repeats compromise the nucleocytoplasmic transport of this important transcription factor TFEB has yet to be elucidated.

The SIGMAR1 plays a key role in facilitating autophagy [67–69], but the exact molecular mechanism remains unknown. Here, we show that the SIGMAR1 formed a complex with POM121. POM121 dysfunction has been shown by several key reports to be a critical pathogenic event in C9ALS-FTD [13,65,70]. In particular, the reduction of POM121 levels has been observed in C9ALS-FTD iPS-derived neurons and postmortem tissues [13]. By associating with POM121, SIGMAR1 chaperoned POM121 to recruit KPNB1 and facilitate the nucleocytoplasmic transport of TFEB. Transport of TFEB into the nucleus is necessary to initiate autophagy and enhance survival in the NSC34 cells. We utilized the SIGMAR1-HSPA5/BiP dissociation assay (a validated SIGMAR1 functional assay) to demonstrate that pridopidine acted as a potent SIGMAR1 agonist, exhibiting the expected biphasic response curve [28]. Pridopidine enhanced the chaperone activity of the SIGMAR1 and ameliorated the TFEB transport deficit in the NSC34 cells expressing HRE. These results suggest that nucleocytoplasmic transport is a tightly orchestrated signaling event in which a ligand-regulated molecular chaperone at the nuclear pore activates a nucleoporin at the central pore to trigger the inbound nucleocytoplasmic transport of important transcription factors. Further, our results also suggest a pharmacological approach targeting the SIGMAR1 for therapeutic development of diverse diseases in which autophagy is impaired.

## Results

### SIGMAR1/Sigma-1 receptor interacted with nucleoporin POM121 and KPNB1/importinβ1 in NSC34 cells

**Figure 1.**
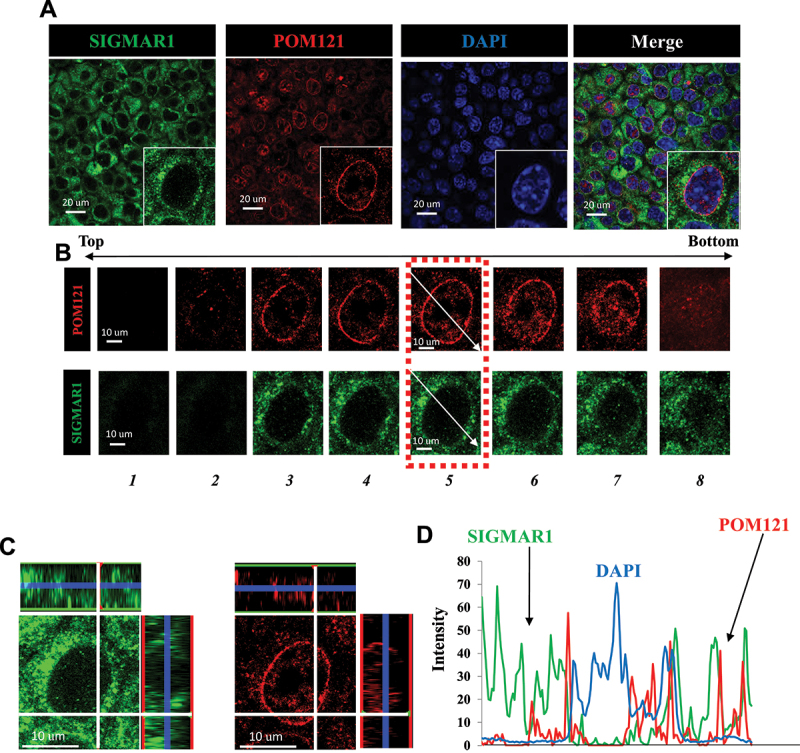
SIGMAR1/Sigma-1 receptor colocalized with POM121 at nuclear envelope in NSC34 motoneuron-like cells. (**A**) SIGMAR1 colocalized with POM121. Confocal images demonstrated the aerial view for the colocalization of SIGMAR1 (green) and POM121 (red) in NSC34 cells. (**B**) Confocal images with multiple Z-axis sections of a chosen cell from (A). (**C**) Confocal images of “Z-axis section 5” in aerial view (middle) and in 3D views (XZ- or YZ- axis on top and right side of the main image) indicating the colocalization of immunoreactive SIGMAR1 (green) and POM121 (red) even on the Z-dimension. (**D**) Tracking (arrows shown in B) of the signal intensities for SIGMAR1, POM121, and DAPI in the “Z-axis section 5” indicated the colocalization of SIGMAR1 and POM121 in close proximity to the nucleus per DAPI staining.

Immunocytochemistry indicated the colocalization of SIGMAR1 and POM121 in NSC34 motor neuron-like cells. The aerial view of SIGMAR1 and POM121 suggested the existence of both proteins at nuclear membrane in proximity to DAPI staining (Figure 1(a)). A cell from Figure 1(a) was chosen (magnified at lower left corner) for the following analyses. Confocal images with Z-axis sections of the cell from Figure 1(a) were obtained (Figure 1(b)). “Z-axis section 5” thus obtained was further examined. The aerial view and two side-views at 90-degree angle, respectively, of the colocalization of SIGMAR1 and POM121 on Z-axis section 5 are shown (Figure 1(c)), indicating that the colocalization of SIGMAR1 and POM121 was not only on the ariel view but also on the Z-dimension. The tracking of the three fluorescence profiles on Z-axis section 5, along the indicated arrows in Figure 1(b), confirmed the colocalization of SIGMAR1 and POM121 at nuclear membrane in close proximity to DAPI staining (Figure 1(d)). Note: The immunocytochemistry here utilized paraffin-embedded sections prepared from cultured NSC34 cells (see Materials and Methods) because the anti-SIGMAR1 antibody (Santa Cruz Biotechnology, B1 anti-SIGMAR1 monoclonal) used in the immunostaining was reported to exhibit the best specificity when tissue samples were embedded in paraffin [71]. The validation of the specificity of the utilized antibody against SIGMAR1 or POM121, respectively, is shown in the Fig. S1 for SIGMAR1 and Fig. S2 for POM121.

**Figure 2.**
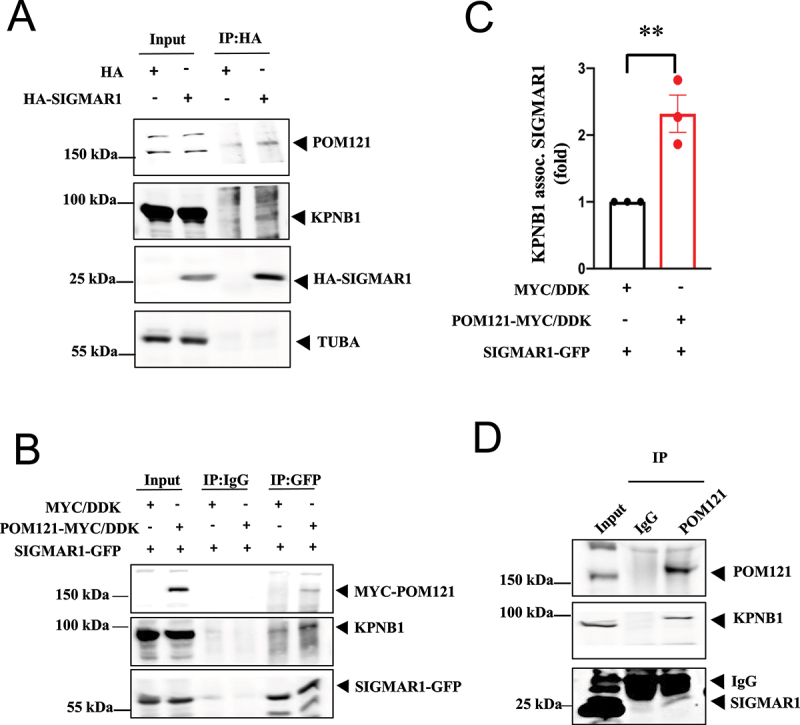
SIGMAR1/Sigma-1 receptor, POM121, and KPNB1/importinβ1 formed a complex. (**A**) In HA-SIGMAR1 transfected NSC-34 cells, coimmunoprecipitation (coIP) study showed the association of HA-SIGMAR1 with POM121 and KPNB1. (**B**) Similarly, in POM121-MYC/DDK and SIGMAR1-GFP co-expressing NSC-34 cells, sample blot demonstrates the coIP of MYC/DDK-POM121, SIGMAR1-GFP, and endogenous KPNB1. (**C**) Summary data from (B) show an increased association of endogenous KPNB1 and SIGMAR1-EGFP. Data are presented as means ± SEM; N = 3; two-tailed unpaired Student<apos;>s *t* test, *p* = 0.0091, **p < 0.01. (**D**) Immunoprecipitation with the POM121 antibody successfully coIPed endogenous KPNB1 and SIGMAR1. n = 3 (A), n = 3 (B), and n = 2 (D) independent experiments with similar results each from biologically independent cells or cellular preparations. Note 1: The intense wide band, observed directly above the SIGMAR1 band, was the IgG light chain (labeled in Figure 2D). Note 2: Our lab has extensive experience in evaluating levels of endogenous SIGMAR1 proteins. From our experience, in order to obtain reliable and measurable levels of “endogenous” SIGMAR1 in a co-IP experiment, a high amount of sample in the co-IP is needed. The amount of sample in the input was for illustration purposes and was therefore smaller than the amount employed in the co-IP. For that reason, the POM121 was bigger than in the input.

Co-immunoprecipitation (co-IP) experiments showed the complex formation between HA-SIGMAR1, POM121, and KPNB1 (Figure 2a). Overexpression of POM121-MYC/DDK increased the co-IP between SIGMAR1 and KPNB1 (Figure 2(b,c)). Endogenous SIGMAR1 associated with POM121 and KPNB1 (Figure 2(d)). Note: The big band signal right above the SIGMAR1 is the IgG light chain (marked in Figure 2(d)). IgG light chains, which have a similar M.W. to the SIGMAR1 were recognized by the secondary antibody in the western blot.

### (G4C2)_31_ dissociated the SIGMAR1/Sigma-1 receptor from POM121 and KPNB1/importinβ1

**Figure 3.**
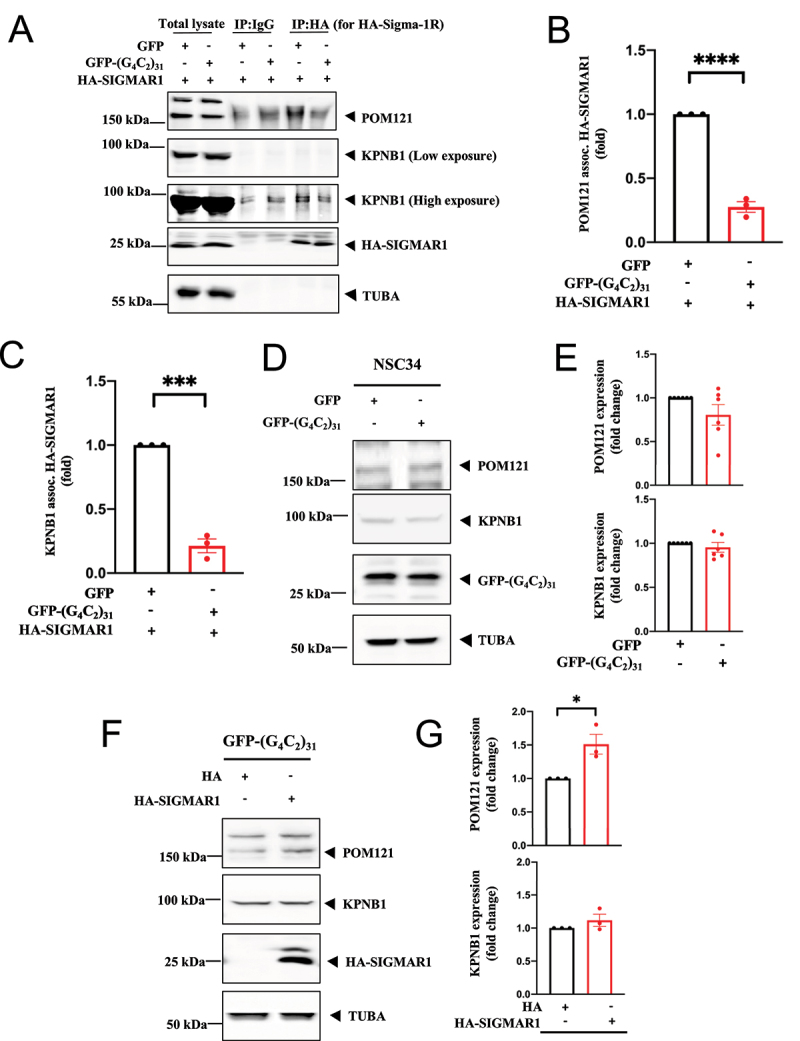
(G_4_C_2_)_31_-RNA repeats attenuated the SIGMAR1/Sigma-1 receptor-POM121 association as well as the SIGMAR1/Sigma-1 receptor-KPNB1/importinβ1 association in NSC34 cell. (**A**) In (G_4_C_2_)_31_-RNA repeats-transfected-NSC34 cells, the HA antibody was used to pull down HA-SIGMAR1. Technically, this is not the HA control. The real control is the IP using IgG in lanes #3, #4, which unfortunately show some background. Western blot showed a decreased association between SIGMAR1 and POM121 as well as between SIGMAR1 and KPNB1. Data were quantified in (**B**) for SIGMAR1-POM121 and in (**C**) for SIGMAR1-KPNB. Data are presented as means ± SEM; N = 3; two-tailed unpaired Student<apos;>s *t* test, ****p <0.0001 (for Pom121) and ***p = 0.0001 (for KPNB1). Note: Band intensities in IgG control [i.e., lanes 3, 4 in (A)] were subtracted from samples lanes [(i.e., lanes numbers 5, 6 respectively in (A)]. (**D**) Sample blot showed POM121 and KPNB1 protein level in EGFP-(G_4_C_2_)_31_-expressing NSC-34 cells. (**E**) Quantification of data from (D) showed no statistically significant difference. Data are presented as means ± SEM; N = 6; two-tailed unpaired Student<apos;>s *t* test, *p* = 0.1308 (for POM121) and *p*= 0.4212 (for KPNB1/importinβ1). (**F**) SIGMAR1 increased POM121 protein expression but not KPNB1 in EGFP-(G_4_C_2_)_31_-expressing NSC-34 cells. (**G**) Quantification of data from (F) showed that POM121 is upregulated by SIGMAR1 overexpression when NSC34 cells were treated with (G_4_C_2_)_31_-RNA repeats. Data are presented as means ± SEM; N = 3; two-tailed unpaired Student<apos;>s *t* test, *p* = 0.0258 (for POM121) and *p* = 0.2765 (for KPNB1), *p < 0.01.

Overnight transfection of NSC34 cells with GFP-(G4C2)_31_ RNA repeats (hereafter referred to as (G4C2)_31_ [4]) caused a significant dissociation of HA-SIGMAR1 from POM121 and KPNB1 (Figure 3(a-c)). Successful transfection of (G4C2)_31_ was validated by the fluorescence in situ hybridization technique. The data showed that G4C2 hexanucleotide foci were formed in the nucleus (Fig. S3).

Transfection of (G4C2)_31_ induced a slight reduction of POM121 levels which did not reach statistical significance (Figure 3(d,e)). Transfection of (G4C2)_31_ did not significantly affect the level of KPNB1 (Figure 3(d,e)). Interestingly, even though overexpressed HA-SIGMAR1 proteins were largely dissociated from POM121 in the presence of (G4C2)_31_ (Figure 3(b)), HA-SIGMAR1 proteins still induced an increase of POM121 (Figure 3(f,g)). The molecular explanation is given below.

We have previously shown that the SIGMAR1 can bind (G4C2) repeats [32]. The observed decrease in POM121 levels suggests that in the presence of (G4C2)_31,_ a large fraction of endogenous SIGMAR1 proteins bound (G4C2)_31_ and might lose their chaperone activity on POM121, thus causing a slight reduction of POM121 (Figure 3(e)). However, when HA-SIGMAR1 proteins were overexpressed, sufficient fraction of HA-SIGMAR1 proteins remained free from binding (G4C2)_31_ and were able to chaperone and increase POM121 levels (Figure 3(a, b,g)). The observation that KPNB1 levels were not affected by (G4C2)_31_ nor by overexpressed HA-SIGMAR1 proteins, suggested that KPNB1 was not a primary target of the SIGMAR1 chaperone and thus remained stable in the presence of the (G4C2)_31_.

These results support a model in which SIGMAR1 proteins bind and chaperone POM121 to recruit KPNB1 for nucleocytoplasmic transport of nucleus-bound cargos including TFEB.

We next examined the selective action of SIGMAR1 on the stability of POM121 and KPNB1.

### SIGMAR1/Sigma-1 receptor stabilized POM121 but not KPNB1/importinβ1 in NSC34 cells

**Figure 4.**
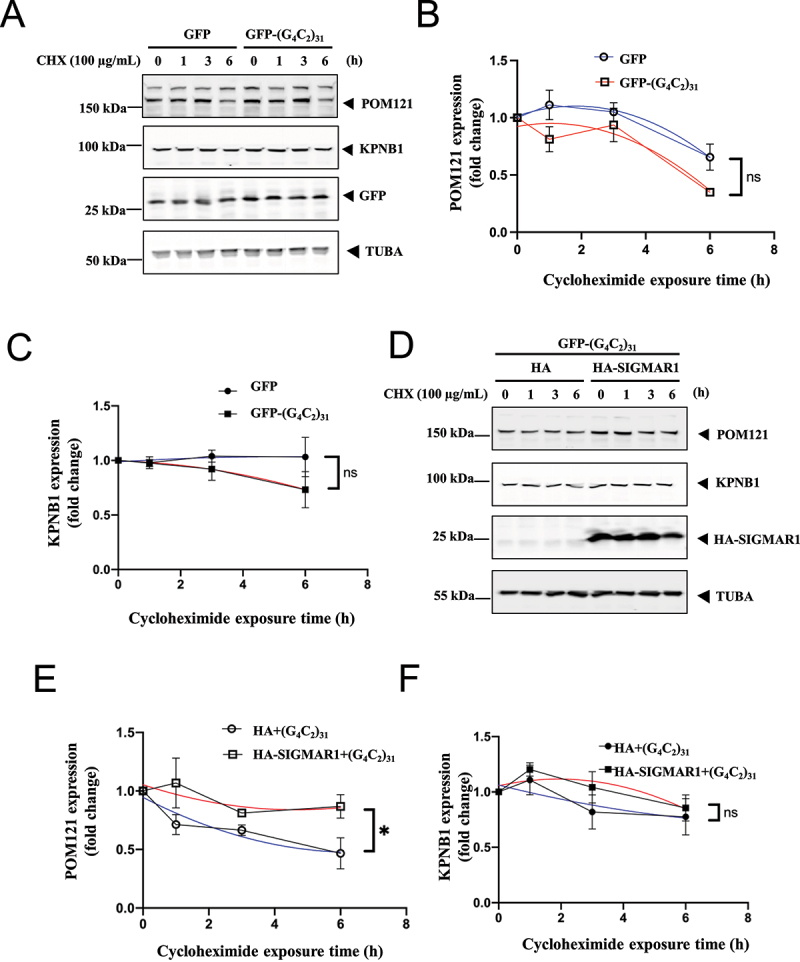
SIGMAR1/Sigma-1 receptor stabilized POM121 but not KPNB1/importinβ1 in (G_4_C_2_)_31_-RNA-treated NSC34 cells. (**A**) Stability of POM121 and KPNB1 in EGFP-(G_4_C_2_)_31_-overexpressing NSC-34 cells in the presence of cycloheximide (100 µg/ml). Time-lapsed levels of POM121 and KPNB1 were examined by western blot. (**B**) Summary data from (A) show a decrease of protein turnover rate in POM121. (**C**) Summary data for KPNB1. Data are mean ± SEM; N = 3; Note: non-liner regression with best fit; for POM121 in (B), *p* = 0.0805; for KPNB1 in (C), *p* = 0.1514. (**D**) Stability of POM121 and KPNB1 in HA-SIGMAR1 and EGFP-(G_4_C_2_)_31_ co-overexpressing NSC34 cells by using the same cycloheximide (100 µg/ml) treatment tracking technique. Time-lapsed levels of POM121 and KPNB1 were examined by western blot. (**E**) Summary data from (D) show a decrease of protein turnover rate in POM121. (**F**) Summary data for KPNB1. Data are means± SEM; N = 3; non-liner regression with best fit; for POM121 (E), *p* = 0.0297, *p < 0.05; for KPNB1 (F), *p* = 0.5202.

Cycloheximide, a known translational inhibitor, was used to inhibit *de novo* protein synthesis and assess the stability of proteins over time. We compared the effect of (G4C2)_31_ on POM121 and KPNB1 stability after 6 h of cycloheximide treatment in NSC34 cells. We observed a small reduction in both POM121 and KPNB1 stability in (G4C2)_31_-transfected cells compared to control cells, which were not statistically significant. Overall, nonlinear regression with best fit showed a slight but not statistically significant reduction in the stability of both POM121 (*p* = 0.0805) and KPNB1 (*p* = 0.1514), when compared to controls (Figure 4(a-c)). Thus, (G4C2)_31_ did not significantly affect POM121 and KPNB1 turnover.

However, overexpression of HA-SIGMAR1 in the presence of (G4C2)_31_, significantly stabilized POM121, but not KPNB1 (p < 0.05, Figure 4d-f).

### Overexpression of either SIGMAR1/Sigma-1 receptor or POM121 restored TFEB and KPNB1/importinβ1 nuclear levels and rescued decreased autophagy in NSC34 cells expressing (G4C2)_31_-RNA repeats

**Figure 5.**
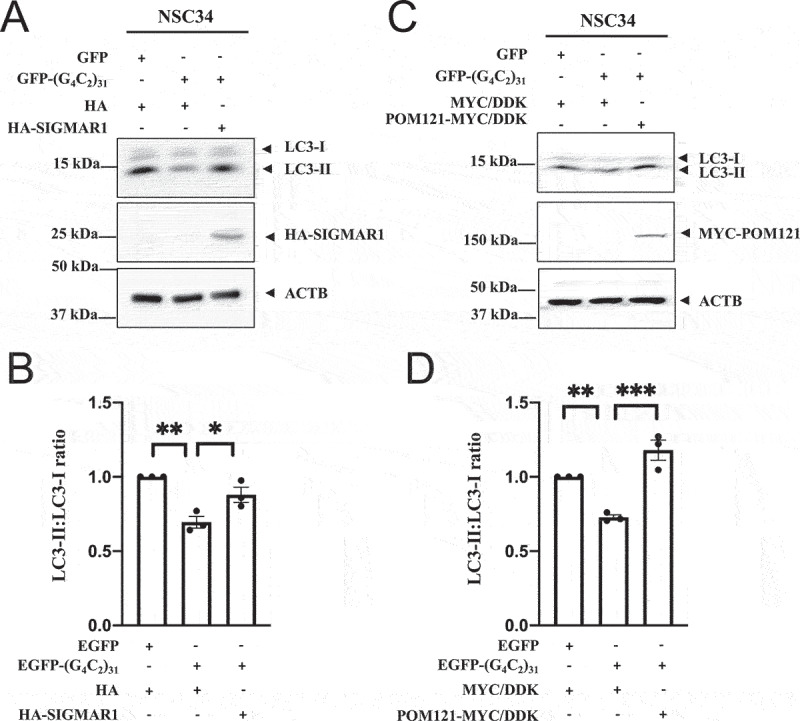
Overexpression of SIGMAR1/Sigma-1 receptor or POM121 in NSC-34 cells rescued (G_4_C_2_)_31_-RNA-repressed autophagy response. (**A**) Overexpression of HA-SIGMAR1 increased the autophagy marker LC3-II in EGFP-(G_4_C_2_)_31_-treated NSC34 cells. (**B**) Quantitative data from (A) are mean ± SEM; N = 3; one-way ANOVA followed by Tukey<apos;>s multiple comparisons test, *p* = 0.0027 and 0.0289 for HA/EGFP and HA-SIGMAR1/EGFP-(G_4_C_2_)_31_ vs HA/EGFP-(G_4_C_2_)_31_, respectively; *p < 0.05, **p < 0.01. Blots were washed 3 times for 10 min with TBST and developed by using the Azure Biosystem c600 Gel Imaging System. The band intensity was analyzed by Image Studio Lite (LiCor 5.2) according to the manufacturer<apos;>s manual. Note: Band intensities were normalized to that of ACTB/β-actin. (**C**) Overexpression of POM121-MYC/DDK increases LC3-II expression. (**D**) Quantitative data from (C) are mean ± SEM; N = 3; one-way ANOVA followed by Tukey<apos;>s multiple comparisons test, *p* = 0.0057 and *p* = 0.0005 for MYC/DDK/EGFP vs MYC/DDK/EGFP-(G_4_C_2_)_31_ and MYC/DDK/EGFP-(G_4_C_2_)_31_ vs POM121-MYC/DDK/EGFP-(G_4_C_2_)_31_, respectively; **p < 0.01, ***p < 0.001. Blots were washed 3 times for 10 min with TBST and developed by using the Azure Biosystem C600. The band intensity was analyzed by Image Studio Lite (LiCor 5.2) according to the manufacturer<apos;>s manual. Band intensities were normalized to that of ACTB/β-actin.

An increase in LC3-II levels as a lipidated product of LC3-I is a well-recognized marker for autophagy [72], and increased levels of LC3-II correspond with enhanced autophagy in NSC34 cells [73]. We therefore used the ratio of LC3-II over LC3-I as a marker of autophagy in our study.

(G4C2)_31_ expression significantly reduced the LC3-II:LC3-I ratio in NSC34 cells (Figure 5(a,b), also in 5C, 5D). However, overexpression of either HA-SIGMAR1 (Figure 5(a,b)) or POM121-MYC (Figure 5(c,d)) significantly restored the reduced LC3-II:LC3-I ratio caused by (G4C2)_31_ (p < 0.05 in Figure 5(b); p<0.001 in Figure 5d).

TFEB is transported from the cytosol through the nuclear pore into the nucleus by KPNB1. This process is crucial for the initiation of autophagy [61,62,65,66,74].

**Figure 6.**
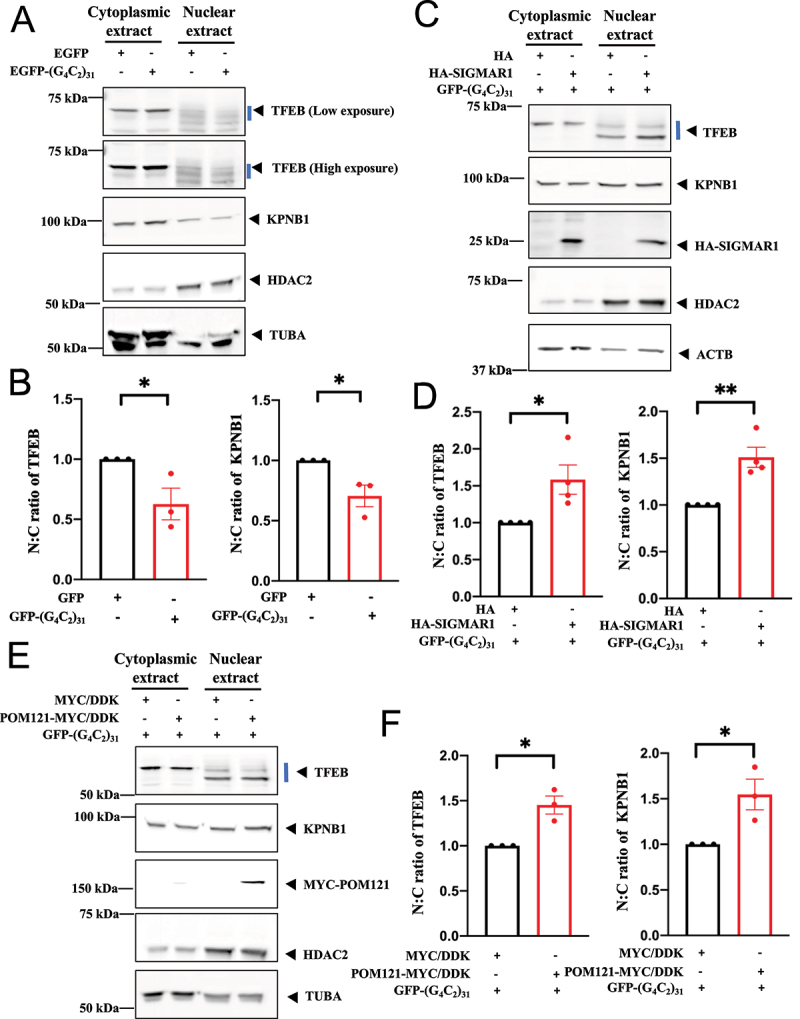
Nuclear to cytosolic ratio (N:C) of TFEB and KPNB1/importinβ1 was decreased by (G_4_C_2_)_31_-RNA repeats while the overexpression of SIGMAR1/Sigma-1 receptor or POM121 rescued the N:C ratio deficit of TFEB and KPNB1/importinβ1 caused by the RNA repeats. (**A**) Overexpression of EGFP-(G_4_C_2_)_31_ in NSC-34 cells decreased the N:C ratio of TFEB and KPNB1. Note: Three repetitions of Figure 6A, are detailed (Fig. S4). (**B**) Quantitative data from (A) are means ± SEM; N = 3; two-tailed unpaired Student<apos;>s *t* test, *p* = 0.0474 (TFEB) and *p* = 0.0307 (KPNB1), *p < 0.05. (**C**) Overexpression of HA-SIGMAR1 rescued the N:C ratio deficit of TFEB and KPNB1 caused by the EGFP-(G_4_C_2_)_31_. Note: Four repetitions of Figure 6C are shown (Fig. S5). (**D**) Quantitative data from (C) are means ± SEM; N = 4; two-tailed unpaired Student<apos;>s *t* test, *p* = 0.0259 (TFEB) and *p* = 0.0032 (KPNB1), *p < 0.05, **p < 0.01. (**E**) POM121-MYC/DDK overexpression rescued the N:C ratio deficit of TFEB and KPNB1 imposed by and EGFP-(G_4_C_2_)_31_. Note: Three repetitions of Figure 6E are shown (Fig. S6). (**F**) Quantitative data from (E) are means ± SEM; N = 3; two-tailed unpaired Student<apos;>s *t* test, *p* = 0.0107 (TFEB) and *p* = 0.0313 (KPNB1), *p < 0.05. Note: The subcellular fraction was conducted by using HDAC2 as nuclear fraction marker and TUBA/α-tubulin or ACTB/β-Actin as cytoplasmic fraction marker throughout the experiments.

**Figure 7.**
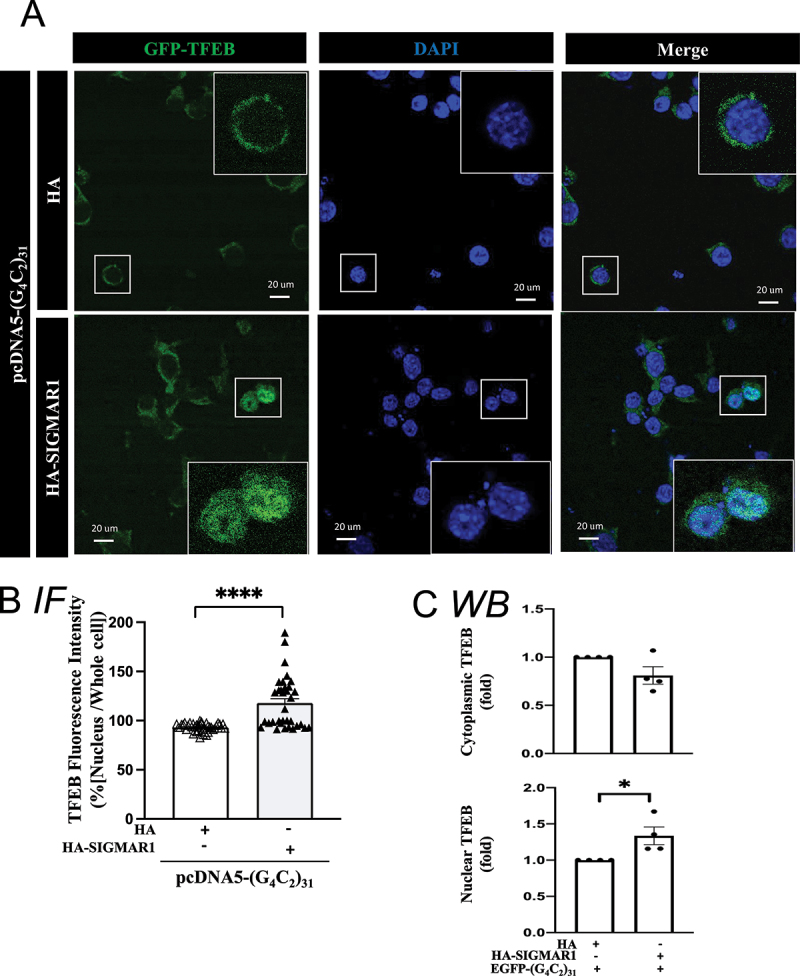
Overexpression of SIGMAR1/Sigma-1 receptor increased nuclear TFEB level in (G_4_C_2_)_31_-RNA repeat-transfected NSC34 cells. (**A**) TFEB translocation in nucleus under SIGMAR1-expressing in (G_4_C_2_)_31_-RNA repeat NSC-34 cells. Confocal images demonstrated GFP-TFEB colocalizes with DAPI in NSC34 cells. (**B**) The quantification data from (A) showed an increased nuclear GFP-TFEB intensity. Intensity analyses were performed by using NIH ImageJ. (version 1.51b). Note: Data shown are percentages of “Average nuclear fluorescence intensity/Average whole cell fluorescence intensity” for each group. HA groups N = 42; HA-SIGMAR1 groups N = 34; two-tailed unpaired Student<apos;>s t test, ****p < 0.0001. (**C**) Overexpression of HA-SIGMAR1 increased the nuclear TFEB expression caused by the EGFP-(G4C2)_31_. Analyses of Figure 6C western blot showed that the overexpression of HA-SIGMAR1 increased the protein level of nuclear TFEB and concomitantly decreased the cytoplasmic TFEB caused by GFP-(G4C2)_31_. Quantitative data are means ± SEM; N = 4; two-tailed unpaired Student<apos;>s t test, *p* = 0.0814 (cytosolic TFEB), *p* = 0.0323 (nuclear TFEB), *p < 0.05.

**Figure 8.**
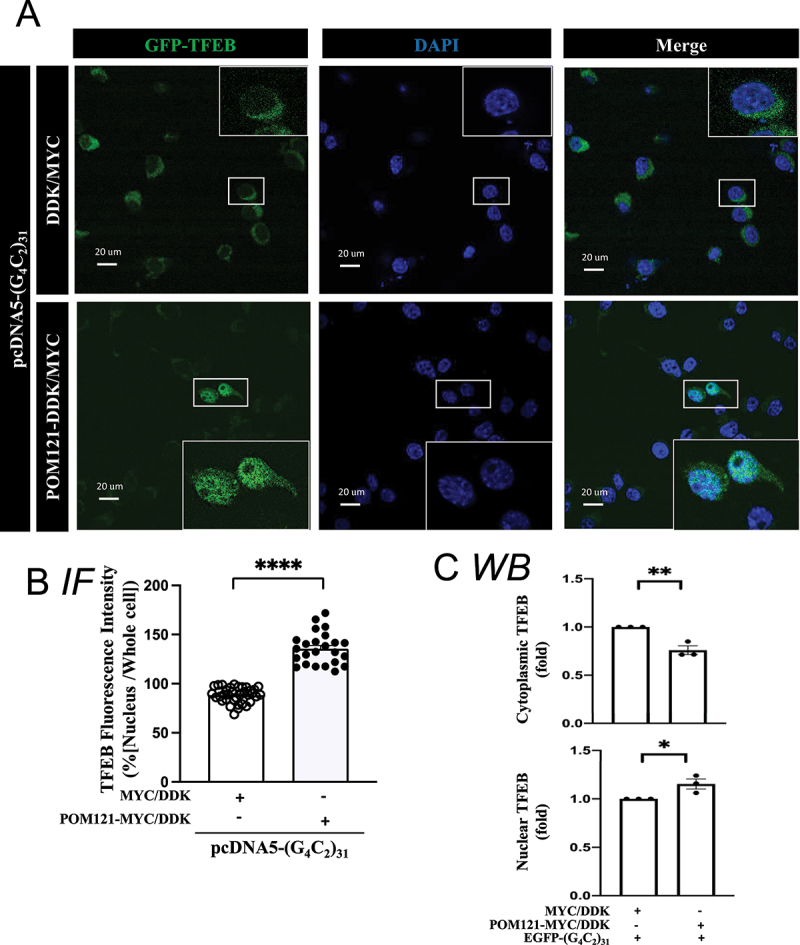
Overexpression of POM121 rescues the TFEB translocation into nucleus in (G_4_C_2_)_31_-RNA repeat-treated NSC34 cells. (**A**) Increased level of nuclear GFP-TFEB in POM121-overexpressing, (G_4_C_2_)_31_-RNA repeat-treated NSC34 cells. Confocal images demonstrated the GFP-TFEB colocalization with DAPI in NSC34 cells. (**B**) The quantification of data from (A) showed a significant increase in the intensity of nuclear GFP-TFEB. The intensity analysis was performed by using NIH ImageJ. (version 1.51b). Note: Data shown are percentages of “Average nuclear fluorescence intensity/Average whole cell fluorescence intensity” for each group; MYC/DDK group, N = 35; POM121-MYC/DDK group, N = 24; two-tailed unpaired Student<apos;>s t test, ****p < 0.0001. (**C**) Analyses of Figure 6E western blot shows that the overexpression of POM121 increased the protein level of nuclear TFEB and concomitantly decreased the cytoplasmic TFEB caused by GFP-(G4C2)_31_. Quantitative data are means ± SEM; N = 3; two-tailed unpaired Student<apos;>s t test, *p* = 0.0060 (cytoplasmic TFEB), *p* = 0.0407 (nuclear TFEB), *p < 0.05, ** *p* < 0.01.

We examined whether (G4C2)_31_ affects the nuclear levels of TFEB and KPNB1 by measuring the subcellular fractionation of TFEB. Changes in TFEB were presented in two manners: (1) the nucleus:cytosol (N:C) ratio of TFEB (Figure 6) or (2) the level of TFEB examined separately in the cytosol or nucleus (Figure 7(c), 8(c), and 11(c)). Note: TFEB is typically recognized in western blots as a two-band entity (for example [75]). In this report, those two bands are marked by a blue straight line in the western blot whenever TFEB western blot is shown throughout this report.

The N:C ratio of TFEB and KPNB1 were significantly reduced by (G4C2)_31_ in NSC34 cells (Figure 6(a,b); p<0.05). Overexpression of either the SIGMAR1 (Figure 6(c,d)) or POM121 (Figure 6(e,f)) rescued the impaired TFEB and KPNB1 N:C ratio caused by (G4C2)_31._

The TFEB level under different conditions was also examined with immunocytochemistry by utilizing GFP-TFEB [65]. The results showed that the overexpression of HA-SIGMAR1 caused an increase of GFP-TFEB in the nucleus of (G4C2)_31_-transfected cells (Figure 7(a,b); p<0.0001). The increase of nuclear TFEB corresponded with a slight decrease in the cytosolic GFP-TFEB (non-significant, Figure 7(c)). Similar patterns were seen when POM121-MYC/DDK was overexpressed in (G4C2)_31_-treated cells, i.e., the nuclear GFP-TEFB was increased while the cytosolic GFP-TFEB was decreased (Figure 8(a,b); p<0.0001). The increase in nuclear TFEB levels was associated with a decrease in its cytosolic levels (Figure 8(c)).

### Pridopidine, a selective SIGMAR1/Sigma-1 receptor agonist, facilitated the dissociation of SIGMAR1 from HSPA5/BiP and potentiated the chaperone activity of the SIGMAR1/Sigma-1 receptor

To explore the action of pridopidine on the SIGMAR1, we used two previously validated assays. The first was the established cellular assay of SIGMAR1-HSPA5 dissociation to confirm the agonistic activity of pridopidine on the SIGMAR1 [28]. The second assay was a chemical reaction assay, employing purified proteins to assess whether pridopidine potentiated the chaperone activity of SIGMAR1, as measured by the inhibition of aggregation of citrate synthase (CS) [28].

**Figure 9.**
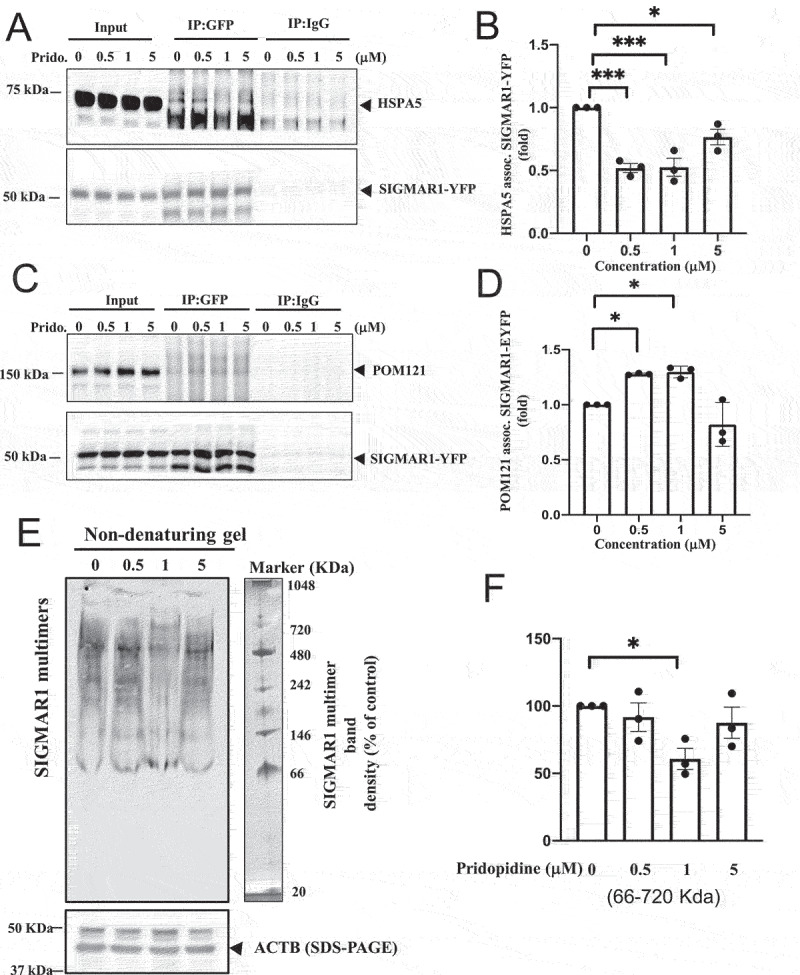
Pridopidine, a highly selective SIGMAR1/Sigma-1 receptor agonist, facilitated the dissociation of SIGMAR1/Sigma-1 receptor from HSPA5/BiP in an apparently biphasic manner which was mirrored by the increased association of SIGMAR1/Sigma-1 receptor with POM121 in a biphasic manner as well as a decreased SIGMAR1/Sigma-1 receptor oligomerization at effective dose. (**A**) Pridopidine caused the dissociation of SIGMAR1 from HSPA5 in an apparently biphasic manner. The three repetitions of the assay detailed in Figure 9A are shown in Figure S7. (**B**) Summary quantitative data from (A) are means ± SEM; N = 3; one-way ANOVA followed by Dunnett<apos;>s multiple comparisons test (HSPA5 assoc. SIGMAR1-EYFP), ***p = 0.0004 (0 µM vs 0.5 µM Pridopidine); ***p = 0.0004 (0 µM vs 1 µM Pridopidine); *p = 0.0279 (0 µM vs 5 µM Pridopidine). Note: Band intensities of HSPA5 were normalized to those of SIGMAR1-YFP. (**C**) Pridopidine conversely increased the association between SIGMAR1 and POM121 in a biphasic manner. (**D**) Summary data from (C) are means ± SEM; N = 3; one-way ANOVA followed by Dunnett<apos;>s multiple comparisons test (POM121 assoc. SIGMAR1-EYFP), *p* = 0.0270 (0 µM vs 0.5 µM Pridopidine); *p* = 0.0194 (0 µM vs 1 µM pridopidine). (**E**) Native gel separation of oligomers of SIGMAR1 proteins. Pridopidine decreased the oligomerization of SIGMAR1 proteins at effective concentration of 1 µM. (**F**) Summary data from (E) indicting the apparent biphasic effect of pridopidine. Data are means ± SEM; N = 3 independent experiments; one-way ANOVA followed by Dunnett<apos;>s multiple comparisons test, *p < 0.05.

Pridopidine acted as a SIGMAR1 agonist, inducing a significant dissociation of SIGMAR1 from HSPA5 in a biphasic manner, with optimal concentrations between 0.5 µM-1.0 µM (Figure 9(a,b)). At a higher concentration (5 µM) pridopidine was less efficacious (Figure 9(a,b)). The agonistic activity of pridopidine was supported by its corresponding potentiation of the association between SIGMAR1 and POM121 in a similar biphasic fashion (Figure 9(c,d). Similar to the results from the SIGMAR1-HSPA5 dissociation assay, 0.5 µM-1.0 µM were the most efficacious doses inducing POM121-SIGMAR1 association (Figure 9(c,d)). These results suggested that pridopidine acted as a SIGMAR1 agonist, causing the dissociation of SIGMAR1 from HSPA5 and allowing the free SIGMAR1 proteins to associate with POM121 and other targets.

SIGMAR1 agonists are known to destabilize the non-active oligomerization state of SIGMAR1 proteins [46,47,76,77]. We therefore examined whether pridopidine reduced the non-active oligomerization state of SIGMAR1 proteins. Indeed, results from experiments employing the native gels showed that pridopidine effectively reduced the oligomerization of SIGMAR1 proteins at 1 µM but not at 5 µM (Figure 9(e,f); p<0.05).

**Figure 10.**
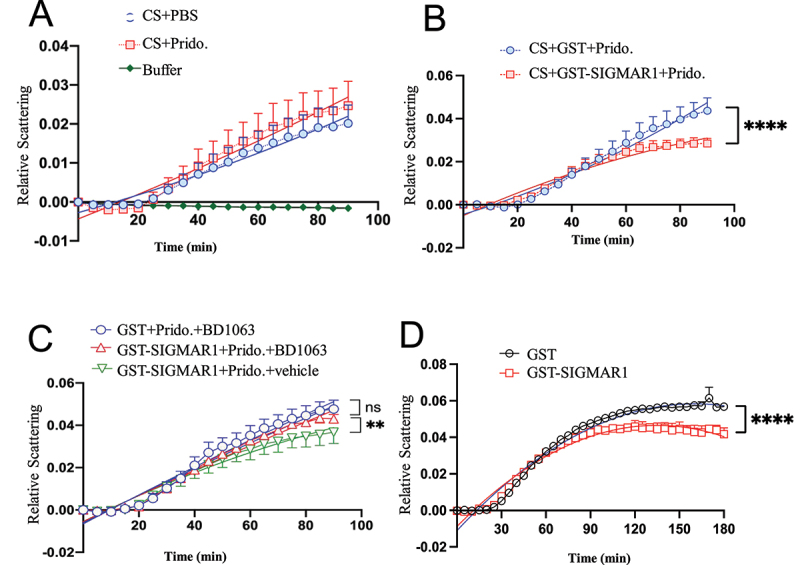
Pridopidine enhanced the SIGMAR1/Sigma-1 receptor chaperone activity in the citrate synthase (CS) aggregation assay, which was inhibited by the SIGMAR1/Sigma-1 receptor antagonist BD-1063. (**A**) Pridopidine by itself did not affect the aggregation of CS. CS (1.1 mM) were incubated at 45°C in the 50 mM HEPES-KOH buffer containing vehicle or pridopidine (200 µM) as shown. The samples were monitored for absorbance at 320 nm, which is indicative of light scattering due to CS aggregation. Relative scattering was expressed in arbitrary units. Data are means ± SEM; N = 3; non-liner regression with best fit, *p* = 0.1008 for CS+PBS vs CS+Pridopidine. (**B**) Pridopidine significantly enhanced the SIGMAR1 chaperoning activity against the aggregation of CS. Data are means ± SEM; N = 3; non-liner regression with best fit; *p* < 0.0001. (**C**) Pridopidine enhancement of SIGMAR1 chaperone activity was inhibited by the SIGMAR1 antagonist BD-1063. Conditions are the same as in (B). BD-1063 was added 10 min before pridopidine. BD1063 (0.2 mM), GST (1 mM), GST-SIGMAR1 (1 mM), pridopidine (200 µM) as shown. Data are means ± SEM; N = 3; non-liner regression with best fit; *p* < 0.001 (GST-SIGMAR1+ Pridopidine+BD1063 vs GST-SIGMAR1+ Pridopidine+vehicle). (**D**) SIGMAR1 by itself was a chaperone in this CS aggregation assay. Non-liner regression with best fit; *p* < 0.0001.

In the chemical reaction assay, pridopidine by itself did not affect the aggregation of CS (Figure 10(a)). Pridopidine, however, significantly (p < 0.0001) potentiated the chaperone activity of SIGMAR1 as demonstrated by the enhancement of SIGMAR1’s anti-aggregation action against CS (Figure 10(b)). The SIGMAR1 antagonist BD-1063 blocked the enhancement by pridopidine on the chaperone activity of SIGMAR1, indicating pridopidine effect was exquisitely mediated by the SIGMAR1 (Figure 10(c)). Interestingly, a previous study shows that C-terminus of SIGMAR1 (a.a. 116–223) acts as a chaperone [28]. We found here that the SIGMAR1 in its full length is also a chaperone (Figure 10(d)).

**Figure 11.**
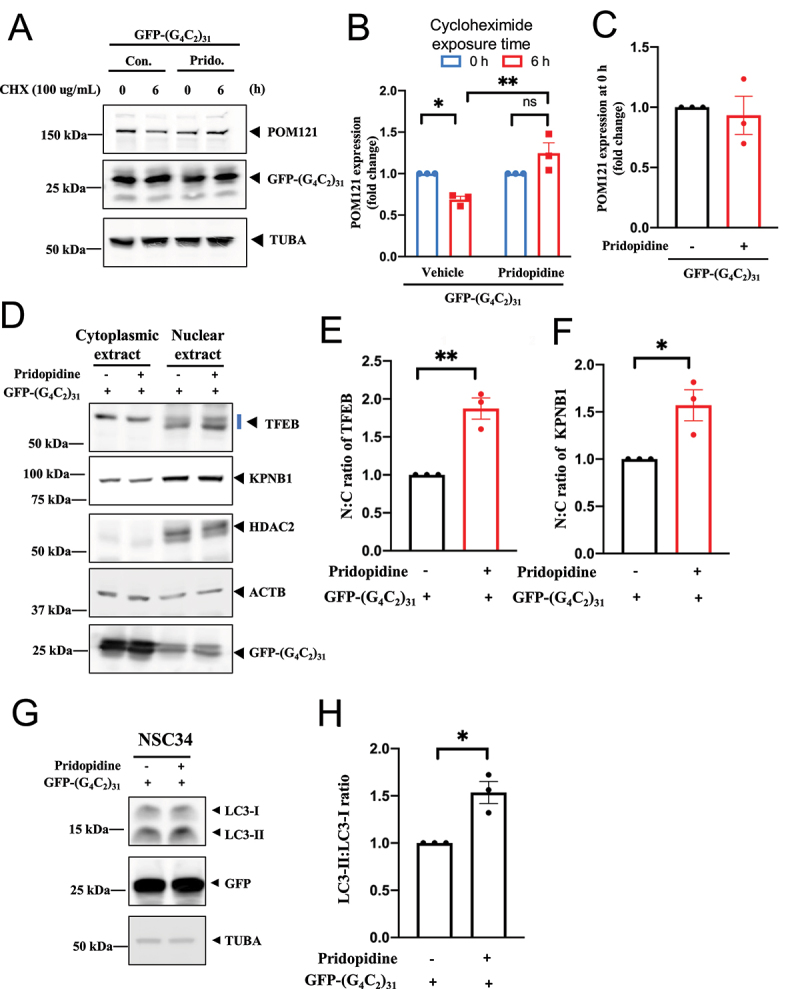
Pridopidine promoted the stability of POM121, increased TFEB and KPNB1/importinβ1 nuclear translocation, and facilitated autophagy in (G_4_C_2_)_31_-RNA-treated NSC-34 cells. (**A**) In the protein turnover experiment using cycloheximide (100 µg/ml) and testing the remaining protein level at the 0- and 6-h time points, pridopidine rescued the decrease of POM121 caused by (G4C2)_31_. Protein levels of POM121 at 0 and 6 h are shown. POM121 protein levels at various time points were then measured by western blot. Note: Cycloheximide was added into culture medium at time zero, i.e., 24 h after the (G4C2)_31_ transfection. (**B**) Quantitative data from (A) are presented. Data are mean ± SEM; N = 3; two-way ANOVA followed by Tukey<apos;>s multiple comparisons test, *p* = 0.0416 for vehicle 0 h vs vehicle 6 h, *p < 0.05; *p* = 0.0015 for vehicle 6 h vs pridopidine 6 h, **p < 0.01. (**C**) The overnight transfection of (G4C2)_31_ did not affect POM121 protein levels at time 0 of the cycloheximide experiment. Data are mean ± SEM; N = 3. (**D**) Pridopidine rescued the N:C ratios of TFEB and KPNB1 as well the level of the autophagy marker LC3-II in NSC34 cells transfected with (G4C2)_31_. A sample western blot is shown. HDAC2 served as nuclear fraction marker and TUBA/α-tubulin as cytoplasmic fraction marker. Three repetitions of the assay from Figure 10D are shown in Figure S8. (**E**) Summary data from (D) are presented where the N:C ratio of TFEB was rescued by pridopidine. Band intensities in cytosol and nuclear extract lanes were normalized to ACTB/β-actin and HDAC2 respectively. (**F**) Summary data from (D) are presented where the N:C ratio of KPNB1 was rescued by pridopidine. Band intensities in cytosol lanes and nucleus lanes were normalized to ACTB/β-actin and HDAC2 respectively. Notes to (E) and (F): The band intensities of the nuclear proteins and cytoplasmic proteins were normalized to different control proteins; Band intensities in cytoplasmic extract were normalized to ACTB/β-actin, while those in the nuclear extract were normalized to HDAC2; The N:C ratio is calculated based on the normalized intensities. Thus, simply looking at the western blot may not reflect the exact N:C ratio. Furthermore, we have used the LiCor 5.2 machine to quantify the bands, which provided high detection sensitivity and accurate quantification of the obtained bands. Data in (E) and (F) are means ± SEM; N = 3; two-tailed unpaired Student<apos;>s *t* test, *p* = 0.0033 (TFEB), **p < 0.01, and *p* = 0.0255 (KPNB1), *p < 0.05. (**G**) Pridopidine rescued LC3-II levels in (G4C2)_31_-transfected NSC34 cells. A sample western blot is shown. Note: Western blots were washed 3 times for 10 min with TBST and developed by using the Azure Biosystem c600. The band intensity was analyzed by Image Studio Lite (LiCor 5.2) according to the manufacturer<apos;>s manual. Band intensities were normalized to that of TUBA/α-tubulin. The N:C ratios were calculated based on the normalized intensities. The three repetitions of the assay from Figure 10G are shown in Figure S9. (**H**) Summary data from (G) are presented as means ± SEM; N = 3; two-tailed unpaired Student<apos;>s *t* test, *p* = 0.0272 for vehicle+EGFP-(G_4_C_2_)_31_ vs Pridopidine+EGFP-(G_4_C_2_)_31_, *p < 0.05.

These results demonstrated that pridopidine was a *bona fide* SIGMAR1 agonist in that it could dissociate the SIGMAR1 from HSPA5, freeing the SIGMAR1 to chaperone target proteins like POM121. Furthermore, pridopidine potentiated the activity of SIGMAR1 when the SIGMAR1 exerted its intrinsic biological action as a molecular chaperone. This study is the first to demonstrate that SIGMAR1 agonists and antagonists affected the chaperone activity of SIGMAR1, and thus provided the first evidence that the SIGMAR1 is a ligand-regulated chaperone.

### Pridopidine enhanced the levels of POM121, TFEB, KPNB1/importinβ1, and LC3-II in NSC34 cells expressing (G4C2)_31._

We next examined the effect of pridopidine on POM121, TFEB, KPNB1, and the autophagy marker, LC3-II:LC3-I ratio, in NSC34 cells expressing the pathogenic (G4C2)_31_.

We evaluated the effect of pridopidine on the reduced stability of POM121 in NSC34 cells, caused by (G4C2)_31._ POM121 stability was assessed by employing cycloheximide in the medium to block *de novo* protein synthesis. In this experiment, NSC34 cells were treated with pridopidine for 1 h before overnight transfection with (G4C2)_31_. POM121 protein levels at various time points were then measured by western blot. NSC34 cells transfected with (G4C2)_31_ showed a significant reduction of POM121 stability after 6 h of cycloheximide treatment (Figure 11(a-b). Pridopidine treatment significantly rescued this reduction of POM121 stability (p < 0.05, Figure 11(a-b)). (G4C2)_31_ transfection had no effect on POM121 levels at the time of pridopidine treatment (Figure 11(c)).

Pridopidine significantly rescued the nuclear levels of both TFEB and KPNB1 in (G4C2)_31_-transfected NSC34 cells (Figure 11(d-f); p<0.05). Additionally, pridopidine enhanced the autophagy marker LC3-II (Figure 11(g)), which was reduced in (G4C2)_31_-transfected NSC34 cells. Results were quantified as the LC3-II:LC3-I ratio (Figure 11(h)).

**Figure 12.**
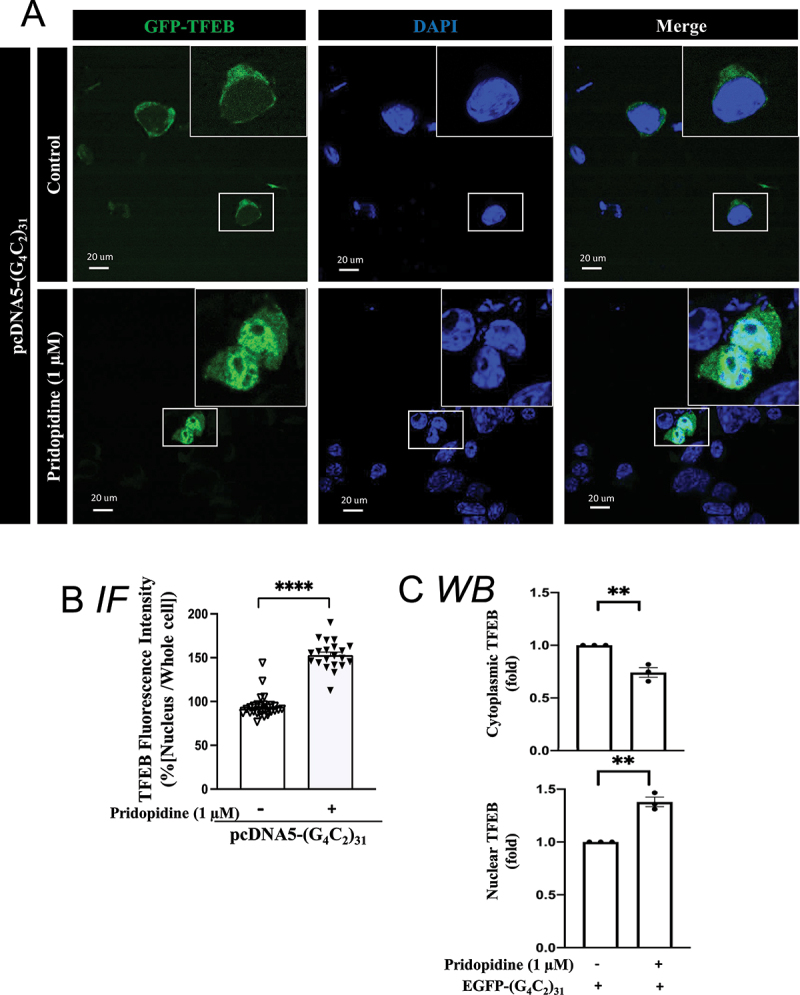
Pridopidine treatment rescued nuclear TFEB level in (G_4_C_2_)_31_-RNA repeats-treated NSC34 cells. (**A**) Nuclear GFP-TFEB level was increased by pridopidine in (G_4_C_2_)_31_-RNA repeated-NSC34 cells. Confocal images demonstrate the GFP-TFEB colocalization with DAPI in NSC34 cells. (**B**) The quantification data from (A) showed an increased intensity of GFP-TFEB in the nucleus. Intensity analysis was performed by using NIH ImageJ. (version 1.51b). Note: Data shown are percentages of “Average nuclear fluorescence intensity/Average whole cell fluorescence intensity” for each group. Control groups, N = 28; pridopidine treatment groups, N = 21; two-tailed unpaired Student<apos;>s t test, *p* = 0.0012, ****p < 0.0001. (**C**) Analyses of Figure 11D shows that pridopidine treatment increased the nuclear TFEB protein expression and decreased the cytoplasmic TFEB caused by the GFP-(G4C2)_31_. Quantitative data are means ± SEM; N = 3; two-tailed unpaired Student<apos;>s t test, *p* = 0.0050 (cytoplasmic TFEB), *p* = 0.0011 (nuclear TFEB), ** *p* < 0.01.

The effect of pridopidine on the nuclear level of TFEB was also examined histologically by utilizing GFP-TFEB as previously reported [65]. Pridopidine significantly increased nuclear levels of TFEB (Figure 12(a,b); p<0.0001). Pridopidine-induced increase in nuclear TFEB levels corresponded with a decrease of its cytosolic levels (Figure 12(c)).

### The effect of (G4C2) repeat length on toxicity in NSC34 cells

**Figure 13.**
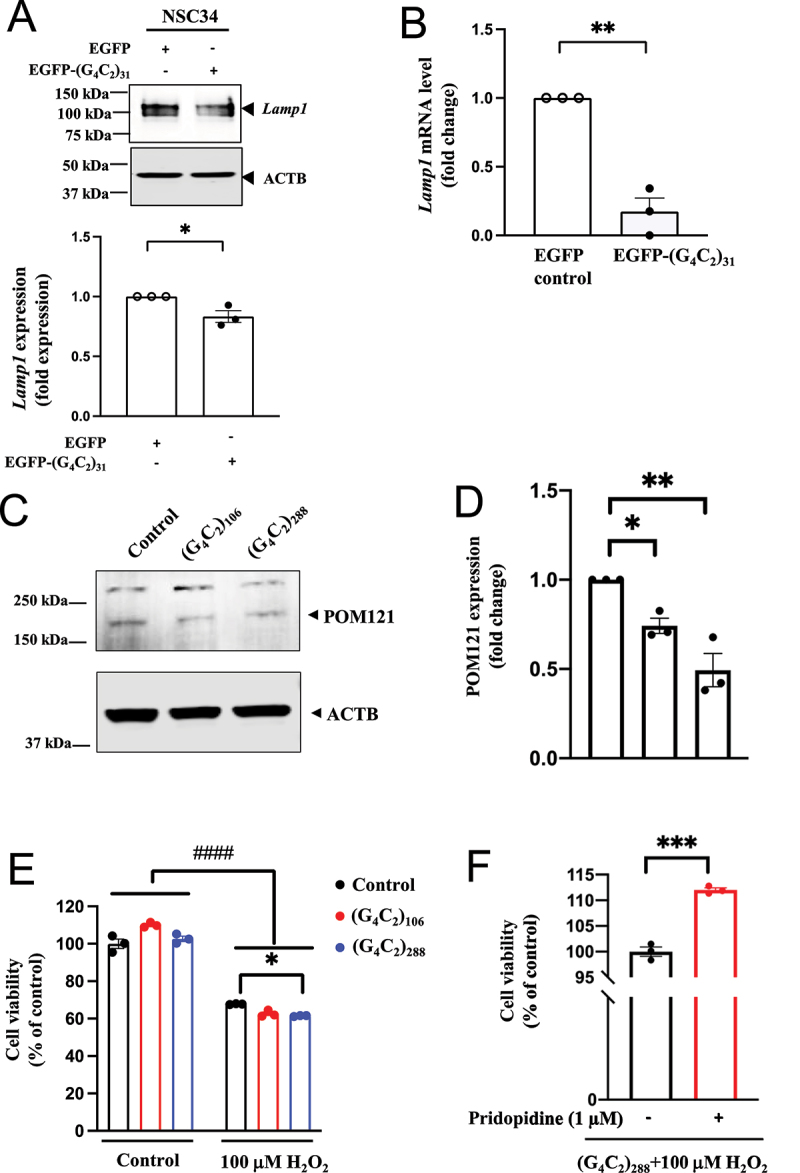
(G_4_C_2_)_31_-RNA repeats reduced *Lamp1* level; (G4C2)_106_- and (G4C2)_288_-RNA repeats attenuate POM121 expression and exacerbated lethality in H_2_O_2_-treated NSC34 cells. (**A**) and (**B**) Overexpression of (G_4_C_2_)_31_-RNA repeat reduced the lysosomal marker LAMP1/*Lamp1* at the protein expression level and the mRNA level in NSC34 cells. Data represent means ± SEM; N = 3; two-tailed unpaired Student<apos;>s t test, *p* = 0.0281 *p < 0.05 (for LAMP1 protein expression) and *p* = 0.0011 (for *Lamp1* mRNA level), **p < 0.01. (**C**) Longer RNA repeats (G_4_C_2_)_106_ and (G4C2)_288_ both significantly decreased POM121 protein expression. (**D**) Quantification of data from (C) showed statistically significant difference. Data are/ presented as means ± SEM; N = 3; One-way ANOVA with Dunnett<apos;>s multiple comparison test, F_(2,6)_ = 18.40, p = 0.0028; *p* = 0.0372, *p < 0.05 (for control and (G_4_C_2_)_106_-RNA repeat); *p* = 0.0016, **p < 0.01 (for control and (G_4_C_2_)_288_-RNA repeat). (**E**) (G_4_C_2_)_288_-RNA repeat promoted H_2_O_2_-induced NSC34 cell death. Data are presented as means ± SEM; N = 3; Two-way ANOVA with Tukey<apos;>s multiple comparison test, Interaction: F_(2,12)_ = 17.55, *p* = 0.0003; H_2_O_2_: F_(1,12)_ = 1457, *p* < 0.0001; G4C2: F_(2,12)_ = 5.512, *p* = 0.0200, *p* < 0.05; ^####^*p* < 0.0001 (for control and H_2_O_2_); *p* = 0.0429, *p < 0.05 (for control+H_2_O_2_ and (G_4_C_2_)_288_-RNA repeat+ H_2_O_2_). (**F**) Pridopidine treatment reverses the H_2_O_2_ toxicity in (G_4_C_2_)_288_-RNA repeats-treated NSC34 cells. Data are presented as means ± SEM; N = 3; two-tailed unpaired Student<apos;>s t test, *p* = 0.0003, ***p < 0.001.

TFEB is a well-known master regulator of lysosomal biogenesis [62]. Lamp1 is a lysosomal membrane protein often used as a lysosomal marker [78]. The effect of (G4C2)_31_ on the protein and mRNA level of *Lamp1* was examined. (G4C2)_31_ decreased the protein level and the mRNA level of *Lamp1* (Figure 13(a,b)).

(G4C2)_106_ and (G4C2)_288_, but not (G4C2)_31_, have been shown to cause a detrimental reduction of POM121 levels in control, non-ALS-FTD iPSNs [13]. Importantly, POM121 levels are known to be reduced both in C9ALS-FTD iPS-derived neurons and in postmortem tissues [13]. Consistent with these data, we found that both (G4C2)_106_ and (G4C2)_288_ induced a significant decrease in POM121 protein levels, with a stronger decrease induced by the 288 repeat compared to the 106 repeat (Figure 13(c,d)). Together, these data suggest that the G4C2 repeat toxicity on POM121 depends on the repeat length.

(G4C2)_106_ and (G4C2)_288_ exacerbate the toxicity of glutamate in *C9orf72* iPSNs, with no effect on the survival of control cells without glutamate treatment [13]. We therefore tested the effects of (G4C2)_106_ and (G4C2)_288_ on toxicity induced by H_2_O_2_ at 100 µM. In the absence of H_2_O_2_, (G4C2)_106_ or (G4C2)_288_ did not affect the survival of NSC34 cells (Figure 13(e), left three graph bars). In the presence of H_2_O_2_, both of the (G4C2) repeats tended to exacerbate the toxicity of H_2_O_2_, but only the effect of (G4C2)_288_ was statistically significant (p < 0.05) (Figure 13(e), right three graph bars). Thus, extended (C4G2) repeats increased the susceptibility of NSC34 cells to H_2_O_2_-induced toxicity, similar to the effect observed in human c9orf72 neurons under the insult of glutamate [13]. Importantly, pridopidine demonstrated a significant protective effect of NSC34 cells against H_2_O_2_-induced toxicity imposed by (G4C2)_288_ (Figure 13(f); p<0.001).

### Schematic summary of the signaling mechanism

**Figure 14.**
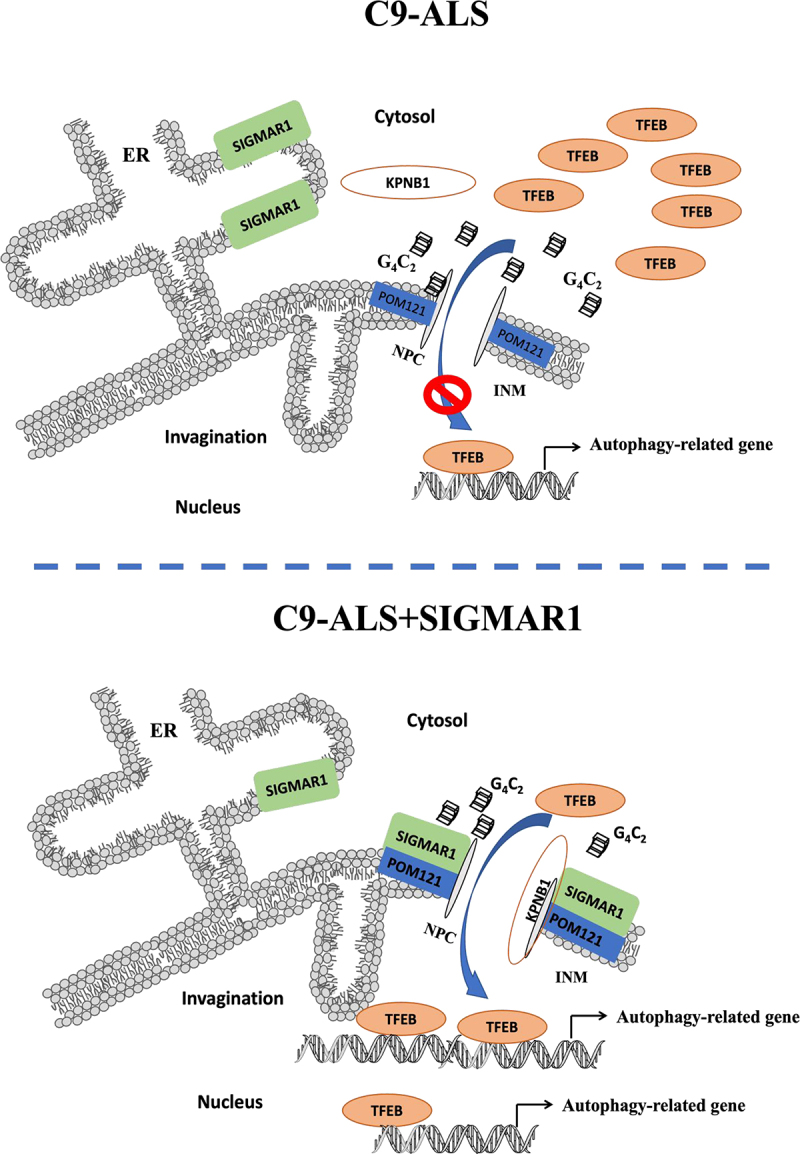
Schematic illustration of the model of the signaling mechanism. SIGMAR1/Sigma-1 receptor translocates from the endoplasmic reticulum (ER) to nuclear membrane when cells are under stressful conditions [83]. (**A**) When motor neurons are under the insult of toxic (G4C2)RNA repeats upon their nuclear pore proteins [13], POM121 cannot recruit KPNB1/importinβ1 for the nuclear import of transcription factor TFEB. (**B**) Sensing such an insult by (G4C2)RNA repeats through an as yet unknown mechanism, SIGMAR1/Sigma-1 receptor proteins move to the nuclear pore to chaperone POM121 to restore its recruitment of KPNB1/importinβ1 for a proper nucleus-inbound cargo transport of TFEB to initiate autophagy for survival of neuron. The SIGMAR1/Sigma-1 receptor agonist pridopidine facilitates this action of SIGMAR1/Sigma-1 receptor.

A schematic summary of the proposed signaling events is shown in Figure 14.

## Discussion

C9orf72 ALS-FTD is a deleterious disease with no effective treatment to date. The key role of the SIGMAR1/Sigma-1 receptor in ALS is demonstrated in human genetic studies showing that an autosomal recessive juvenile form of ALS is with the loss-of-function E102Q mutation of SIGMAR1 [79], while patrial loss-of-function mutations in SIGMAR1 cause an adult-onset ALS, demonstrating a dose-relation between SIGMAR1 function and disease severity. Furthermore, several reports suggest that SIGMAR1 agonists exert beneficial neuroprotective effects in ALS cellular and mouse models [33]. Taken together, these data point out a putative role of SIGMAR1 in ALS etiology. We previously reported that the SIGMAR1 chaperone can attenuate the insult of the HRE in both cellular and *Drosophila* models of ALS-FTD. We further demonstrated that the SIGMAR1 chaperones NUPs 358, 214, and 50, and reduces the deleterious HRE at the NP, ensuring proper functioning of RAN GTPase as an energy source for the transportation of cargos [32].

Here, we show that the SIGMAR1 chaperone played an additional key role attenuating HRE toxicity by facilitating the nucleocytoplasmic transport of TFEB (a key modulator of the initiation of autolysosomal function) and promoting autophagy [61,62,65]. The SIGMAR1 acted by chaperoning POM121, which in turn recruited KPNB1/importinβ1 that carried the TFEB cargo into the nucleus [66]. Thus, in resonance of our recent findings showing that the SIGMAR1 chaperones NUP50, NUP214, NUP358 [32], we provide data here to support a critical role of the SIGMAR1 at the NP modulating nucleocytoplasmic transport. The role of the SIGMAR1, chaperoning and stabilizing the POM121, is of great importance, because POM121 is the gate-keeper ensuring the stability of other NUPs at the NP. Loss of POM121 is a critical pathogenic event in C9ALS-FTD [13].

Our data demonstrate that the SIGMAR1 regulated TFEB transport by functioning as a chaperone of POM121, directly linking the control of nucleocytoplasmic transport by a NUP (POM121) to KPNB1-dependent transport [73]. POM121 and TFEB are negatively affected by HRE [13,65]. The present study provides a potential mechanistic link between reduced POM121 levels and impaired nuclear shuttling of TFEB, in the presence of pathogenic (G4C2)_31_ repeats.

We also evaluated the effects of pridopidine, a potent and selective SIGMAR1 agonist, on the deleterious effects of HRE on nucleocytoplasmic transport. Pridopidine demonstrates neuroprotective effects in preclinical models of several neurodegenerative diseases including ALS, Huntington disease, Alzheimer disease and Parkinson disease [50–57]. These effects are exquisitely mediated by the SIGMAR1, as a genetic deletion or pharmacological inhibition of the SIGMAR1 completely abolishes pridopidine<apos;>s protective effects [50,51,54,56]. Importantly, pridopidine<apos;>s effect adheres to the established biphasic dose response characteristic of SIGMAR1 agonists in preclinical models and in clinical trials [41,58].

Our data provide a potential biological explanation for the observed biphasic dose response of pridopidine. Using the canonical cellular assay [28], we demonstrated that pridopidine facilitated SIGMAR1 dissociation from HSPA5/BiP in a biphasic manner. Pridopidine concentrations of 0.5 µM and 1 µM caused a significant ~50% dissociation of SIGMAR1 from HSPA5, freeing the SIGMAR1 to interact with its target proteins such as POM121. A higher concentration of 5 µM was less efficacious showing ~30% dissociation. This effect was mirrored by pridopidine-induced interaction of SIGMAR1 and POM121. Pridopidine at 0.5 and 1 µM showed a significant effect, enhancing SIGMAR1-POM121 interaction while the higher concentration of 5 µM had no effect (Figure 6(a-d)). Similar apparently biphasic manner was also seen in the pridopidine ability to attenuate the oligomerization of SIGMAR1 proteins. Pridopidine at 1 µM demonstrated a significant effect reducing SIGMAR1 oligomerization, while 0.5 and 5 µM were less efficacious (Figure 6(e,f)). These findings highlight the importance of appropriate dosing to establish an optimal dose to be used in the clinical development of SIGMAR1 agonists.

The present study provides the first experimental evidence to support the SIGMAR1 as a ligand-regulated chaperone. We previously showed that the SIGMAR1 protein *per se* can chaperone its client against aggregation [28]. However, there is no study to date that shows this chaperoning activity of SIGMAR1 is regulated by ligands, in particular in an agonist/antagonist fashion. Here, using a chemical assay examining the heat-induced aggregation of citrate synthase (CS), we show that pridopidine acted as an agonist by potentiating the chaperone activity of SIGMAR1 against CS aggregation (Figure 6(h,i)). Remarkably, this action of pridopidine was blocked by the SIGMAR1 antagonist BD-1063, confirming pridopidine<apos;>s effect is completely mediated by the SIGMAR1 (Figure 6(i)). Importantly, this assay is of great interest as it can be utilized as a high throughput screening assay to identify new therapeutic agents acting as SIGMAR1 ligands.

It is noteworthy that almost all molecular chaperones depend on ATP. However, in the present study we found that the SIGMAR1 chaperone was an ATP-independent chaperone, similar to a recently discovered ATP-independent chaperone called Spy [80]. Because the SIGMAR1 exists in many different locations within the cell, the ATP-independent nature of the SIGMAR1 chaperone may allow it to function both in the intracellular [28] and extracellular space [81].

We utilized the mouse NSC34 cells to investigate biological mechanisms relevant to C9orf72 ALS/FTD specifically on the HRE toxicity. Overexpression of (G4C2)_31_ RNA repeats did not cause a significant reduction in POM121 levels, which is in agreement with what is reported in the literature [13]. However, a significant reduction in POM121 is observed in human iPSNs as well as in C9orf72 ALS-FTD postmortem tissues. We therefore evaluated the effects of longer, non-fused endogenous (G4C2)_106_ and (G4C2)_288_ RNA repeats on POM121 levels in NSC34 cells. We demonstrated that both (G4C2)_106_ and (G4C2)_288_ induced a significant decrease in POM121 protein levels, with a stronger decrease induced by the longer (i.e., 288) repeat compared to the 106 repeat (Figure 13(c,d)). Furthermore, in agreement with the findings in human neurons, we now demonstrated that POM121 reduction on its own, in NSC34 cells did not lead to neurotoxicity. However, NSC34 cells transfected with either (G4C2)_106_ or (G4C2)_288_ repeats showed increased susceptibility to H_2_O_2_-induced toxicity (Figure 13(e)). These findings again correspond with the findings in human C9orf72 neurons, showing a similar increased susceptibility to glutamate-induced toxicity, providing support for the validity of the NCS34 cells expressing (G4C2)_106_ or (G4C2)_288_ as a cellular model to study the toxicity of HRE which is known to be of utmost importance in C9orf72 ALS-FTD related mechanisms [13].

In summary, the ATP-independent SIGMAR1 chaperone is a critical player in a cell<apos;>s defense against the insult from C9orf72 HRE in ALS-FTD. The SIGMAR1 does so by its innate ability to chaperone the nucleoporin POM121, which in turn recruits KPNB1/importinβ1 to facilitate the nucleocytoplasmic transport of TFEB, a critical transcription factor for initiation of autophagy. The SIGMAR1 selective agonist pridopidine activates the SIGMAR1 by facilitating its dissociation from HSPA5/BiP and enhances the SIGMAR1ʹs innate activity as a chaperone to maintain cellular health against pathological insults like C9orf72 HRE in ALS-FTD.

Most importantly, we show that pridopidine, a potent and selective SIGMAR1 agonist, exerted a significant neuroprotective effect against H_2_O_2_-induced toxicity in NSC34 cells expressing the pathogenic (G4C2)_288_ repeat. Pridopidine is currently in clinical development for the treatment of HD and ALS. Our findings reinforce previous data published by other labs, demonstrating that pridopidine exerts neuroprotective effects in numerous models of neurodegenerative diseases including ALS [54], HD [55,82], PD [53], and Alzheimer disease [52], exclusively mediated via activation of the SIGMAR1, and support the ongoing clinical development of pridopidine for the treatment of ALS.

## Materials and methods

### Cell culture and transfection

The mouse motor neuron (NSC34) cell line was purchased from CELLutions Biosystems INC., CLU140) and Neuro2A cell line was purchased from American Type Cell Collection (CCL-131). Cells were maintained and grown in complete culture Dulbecco<apos;>s modified Eagle<apos;>s medium (DMEM; GIBCO, 11,965–092) containing 10% Fetalgro bovine growth serum (RMBIO, FGR-BBT) and 1% penicillin-streptomycin (GIBCO, 15,140–122). Cell monolayers of 70% density at 10-cm culture dish were used for transfection with plasmids using PolyJet reagent (SignaGen Laboratories, SL100688). In all, the PolyJet reagent and plasmids ratio (2:1) were incubated in 0.5 mL serum-free DMEM for 20 min at room temperature. Subsequently, mixed DNA-polyJet complexes were added into 10-cm culture dish, and then incubated at 37°C in a 5% CO_2_ incubator (Thermo Fisher Scientific) for 24 h.

### Immunostainings

#### Regular procedures (without paraffin embedding of cells)

: NSC34 cells (CELLutions Biosystems INC, CLU140) or Neura2A (American Type Cell Collection, CCL-131) cells were seeded on a glass coverslip overnight at 37°C in an incubator followed by fixation with 4% paraformaldehyde in PBS (137 mM NaCl, 2.7 mM KCl, 8 mM Na_2_HPO_4_, 2 mM KH_2_PO_4_, pH 7.4) at room temperature for 20 min. After washing with PBS three times, cells were incubated with permeabilization buffer (0.1% Triton X-100 [SigmaAldrich, T-9284] in PBS) for 10 min. After washing three times with PBS, slides were incubated with blocking buffer (10% normal goat serum; Abcam, ab7481) at room temperature for 1 h and incubated thereafter with indicated primary antibodies in diluted blocking buffer (1% normal goat serum and 0.1% Triton X-100 in PBS) at proper dilution overnight at 4°C. Cells were then washed three times with wash buffer (0.1% Triton X-100 in PBS) and incubated in PBS with Alexa Fluor 488- (Thermo Fisher, A11092) or Alexa Fluor 568-conjugated secondary antibodies (Thermo Fisher, A11011) for 1 h. Cells were then washed three times with PBS and mounted with Prolong gold antifade mountant with DAPI (Cell Signaling Technology, 4083S). Images of cells were captured by confocal microscopy (Perkin-Elmer Modular laser system 2.0 with Nikon Eclipse TE2000E microscope and Volocity version 6.3 software). Three-dimensional reconstructions were made from the Z-series images. NIH ImageJ was conducted to analyze SIGMAR1/Sigma-1 receptor (green) and POM121 (red) colocalization.

#### Paraformaldehyde-Fixed Paraffin Embedded (PFPE) cells for immunostaining

The procedure for PFPE of NSC34 cells and Neura2A cells was according to a protocol available from the Histology Core of University of Virginia (https://med.virginia.edu/biorepository-and-tissue-research-facility/wp-content/uploads/sites/167/2015/10/Cell-culture-for-FFPE.pdf) with an only modification in this report on the size of the conical centrifuge tube (15 ml instead of 50 ml). Accordingly, the protocol and instructions are given as follows. The volume of the packed cell pellet is approximately 0.5 ml. This requires approximately four 75-cm^2^-sized dishes, or two 150-cm^2^ dishes of near-confluent cell culture. Less material will result in a size-limited preparation. For adherent monolayer cells, trypsinization is not allowed as this may destroy cell-surface protein markers. Working quickly, remove flasks from the incubator, and scrape the cells into the media. Transfer to a sterile 15 ml (the only modification) polypropylene conical centrifuge tube. Spin at room temperature for 5 min in swinging bucket centrifuge (setting 3 for 5 min in a standard clinical centrifuge, or approximately 200 x g). Aspirate media off cell pellet. Very slowly, add 20 ml neutral buffered formalin (NBF; SigmaAldrich, MFCD00003274) (4°C), letting it flow gently down the side of the tube, in order not to disturb the pellet. Re-centrifuge if needed for example if the cell pellet is disturbed. After centrifugation, the supernatant was removed and the pellet was fixed with 10% neutral buffered formalin (NBF; SigmaAldrich, MFCD00003274) overnight at 4°C without disturbing the pellet. It is essential to tap the bottom of the conical tube to allow the pellet to detach from the wall of the tube to allow for a complete assessment of formalin to the whole surface of the pellet. If the delivery of the preparation cannot be made to the PFPE facility within 24 h of the start of fixation, formalin was removed and replaced with 20 ml of 70% ethanol without resuspending the pellet. This acts as a non-crosslinking preservative, and the cells can be kept this way indefinitely at 4°C. Note: Cells thus obtained should not be frozen. Two facilities were chosen in this study (AML Laboratories, St. Augustine, FL; Johns Hopkins Histology Core, Baltimore, MD) to process the cell pellet into a paraffin block that was used for sectioning into 5-μm sections on a glass slide for immunostaining.

#### Immunostainings on sections from PFPE NSC34 and Neura2A cells

The cellular sections on slides were deparaffinized in sequels of xylene solutions (5 min for 3 times) followed by rehydration with serial dilutions of ethanol (100% to 70% 5 min each). After washing out ethanol with Milli-Q water (3 min for 3 times), sections underwent antigen-retrieval as follows. Sections were treated with retrieval buffer (10 mM Tris-1 mM EDTA, pH 9.0) for 10 min at room temperature and then were heated in prewarmed retrieval buffer at 95°C for 20 min, followed by post-incubation at room temperature for 30 min. The antigen retrieval was performed in 50 ml polypropylene conical tube. After washing with TBS (20 mM Tris and 157 mM NaCl, pH 7.4; 3 min for 3 times), sections were blocked with 10% normal goat serum (Abcam, Ab7481) and 1% BSA (SigmaAldrich, A7030) in TBS containing 0.1% Tween20 (v/v; TBST) for 1 h at room temperature. The cellular sections then underwent a 2^nd^ blocking with goat anti-mouse IgG (Fc fragment specific [JacksonImmnoResearch Lab, 115–001-008]) for 1 h at room temperature. After washing with TBS (3 min for 3 times), sections were incubated with the mouse anti-SIGMAR1/Sig1R (B5, 1: 220x; Table 1), rabbit anti-POM121 (1: 100x; Table 1), mouse IgG antibody (Table 1), or rabbit IgG antibody (Table 1) in the antibody dilutant (5% NGS and 1% BSA in TBST) overnight at 4°C. Following TBST washings (5 min for 4 times), sections were incubated with Alexa Fluor 488-conjugated goat anti-mouse IgG or Alexa Fluor 647-conjugated goat anti-rabbit IgG (1:300x, Table 1) in antibody dilutant for 1 h at room temperature in the dark. Sections were washed with TBST (5 min for 4 times) then counterstained with 4´,6-diamino-2-phenylindole (DAPI; Invitrogen, Cell Signaling Technology, 4083S; 500 ng/mL in TBS) for 10 min at room temperature. Sections were then washed with TBS (5 min for 3 times), mounted with Prolong Diamond Antifade Mountant (Invitrogen, P36961) and covered with glass coverslips. Zeiss confocal microscope LSM 710 system with Zeiss AX10 microscope and Zen version 2.3 SP1 for data acquisition, was used to examine the staining on sections. The Photoshop (Adobe Photoshop CC 2019, version 20.0.10) and NIH ImageJ software were used for images processing subsequent to data acquisition.

**Table 1.** List of antibodies, cDNA plasmid vectors, and oligonucleotide sequences. WB: western blot; IF: immunofluorescence; IP: immunoprecipitation.

Antibodies	Species	dilution ratio; WB; IF; IP	Source	Category No.
ACTB/β-actin	mouse	WB: 1:10000x	Proteintech Group, Inc.	66,009-1-lg
HSPA5/BiP	mouse	WB: 1:1000x	BD Transduction Lab	610,979
GFP	rabbit	WB: 1:1000x; IP: 2 βg	Proteintech Group, Inc.	50,430-2-AP-1
HA	rabbit	WB: 1:1000x; IP: 2 βg	Proteintech Group, Inc.	51,064-2-AP
HDAC2 (3 F3)	mouse	WB: 1:10000x	Cell Signaling Technology	5113S
KPNB1/importinβ1(3E9)	mouse	WB: 1:1000x	ThermoFisher Scientific	MA-3-070
LAMP1/CD107A	rat	WB: 1:1000x	BD Pharmingen	553,792
LC3A/B (D3U4C) XP	rabbit	WB: 1:1000x	Cell Signaling Technology	12741S
MYC (71D10)	rabbit	WB: 1:1000x	Cell Signaling Technology	2278S
POM121 antibody, rabbit polyclonal	rabbit	WB: 1:1000x; IF: 1:100x, 200x	Novus Biologicals	NBP2-19,890
SIGMAR1/Sigma-1 receptor (B-5) antibody	mouse	WB: 1:500x, 1:1000x; IF: 1:200x	Santa Cruz Biotechnology	sc-137,075
TFEB	rabbit	WB: 1:1000x	Bethyl Laboratories	A303-673A
TUBA/αβtubulin	mouse	WB: 1:10000x	SigmaAldrich	T-5168
Albumin, bovine serum			SigmaAldrich	A7030
Normal goat serum			abcam	Ab7481
Normal mouse IgG antibody	mouse	IP: 2 βg	Santa Cruz Biotechnology	sc-2025
Normal rabbit IgG antibody	rabbit	IP: 2 βg	Cell Signaling Technology	2729S
Goat anti-mouse IgG,			Jackson ImmunoResearch Lab.	115–001-008
Fc fragment specific				
Peroxidase-conjugated AffiniPure goat anti-mouse IgG, Fc fragment specific	mouse	WB: 1:10000x	Jackson ImmunoResearch Lab.	115–035-164
Peroxidase-conjugated AffiniPure goat anti-mouse IgG, light chain specific	mouse	WB: 1:10000x	Jackson ImmunoResearch Lab.	115–035-174
Peroxidase AffiniPure goat anti-rabbit IgG, Fc fragment specific	rabbit	WB: 1:10000x	Jackson ImmunoResearch Lab.	111–035-046
IRDye 680RD goat anti-mouse IgG secondary antibody	mouse	WB: 1:10000x	Li-Cor Biosciences	926–68,070
IRDye 800CW goat anti-rabbit IgG secondary antibody	rabbit	WB: 1:10000x	Li-Cor Biosciences	926–32,211
Alexa Fluor 488 goat anti-mouse IgG (H + L)	mouse	IF: 1:300x	Thermo Fisher Scientific	A11092
Alexa Fluor 568 goat anti-rabbit IgG (H + L)	rabbit	IF: 1:300x	Thermo Fisher Scientific	A11011
Alexa Fluor 647 goat anti-rabbit IgG (H + L)	rabbit	IF: 1:300x	Thermo Fisher Scientific	A21245
**Plasmid Vectors**	**Species**	**Source**	**Category Number**	
*POM121* shRNA Plasmid (m)	mouse	Santa Cruz Biotechnology	sc-152,388-SH	
Control shRNA vector		SigmaAldrich	SHC002	
Cy3-(G4C2)4 for RNA FISH test		Integrated DNA Technologies	Custom made	
Two pSpCas9BB-2A-Puro (PX459) plasmids containing CRISPR guide RNA (gRNA) sequences: 5’- GGCCCCGGGCATAGGCCCGA-3’ and 5’-CGCTAGAATGCCGTGGGCCG-3’	mouse SIGMAR1	GenScript	SC1948-459	
pCMV3-HA-SIGMAR1/Sigma-1 receptor	mouse	Sino Biological	MG57873-NY	
pCMV6-MYC/DDK		Origene Technologies Inc	PS100001	
pCMV6-POM121-MYC/DDK	mouse	Origene Technologies Inc	MR211792	
pCMV3-SIGMAR1/Sigma-1 receptor-GFP	mouse	Origene Technologies Inc	MG57873-ACG	
EGFP-(G4C2)31		self-construct; Mauro Cozzolino		
pcDNA5-(G4C2)31		Self-construct; Mauro Cozzolino		
SIGMAR1/Sigma-1 receptor-EYFP	mouse	self-construct; Teruo & Su, 2007 Cell		
EYFP-N-SIGMAR1/Sigma-1 receptor		self-construct; Teruo & Su, 2007 Cell		
pcDNA3.1-(G4C2)106		Mizielinska et al., 2014 Science; gift from Adrian Isaacs		
pcDNA3.1-(G4C2)288		Mizielinska et al., 2014 Science; gift from Adrian Isaacs		
**Oligonucleotides; Primer pairs (5’ to 3’) used for quantitative real-time PCR**				
**Gene name**		**Source**		
mouse *Gapdh*	F: GAGAGGCCCTATCCCAACTC; R:CCGCATTAAAACCAAGGAGA	I Integrated DNA Technologies		
mouse *Lamp1*	F: TCTTCAGTGTGCAGGTCCAG; R: CTGCCAATGAGGTAGGCAAT	I Integrated DNA Technologies		

### Western blot

Transfected cells were harvested and lysed using the modified radioimmunoprecipitation assay (RIPA) lysis buffer (50 mM Tris-HCl, pH 7.4, 150 mM NaCl, 0.05% sodium dodecyl sulfate [SDS], 0.5% Triton X-100, and 0.05% sodium deoxycholate [SigmaAldrich, D6750]) supplemented with EDTA-free protease inhibitor cocktail tablets (Complete Mini, EDTA-free; Roche Diagnostics, 11,836,170,001) on ice for 30 min. Further, an equal amount of proteins was denatured with SDS 4X sample buffer (Bio-Rad, 161–0747) containing 1% 2-mercaptoethanol and heated at 95°C for 10 min. These protein samples were separated by using SDS-polyacrylamide gel electrophoresis (SDS-PAGE) and transferred onto a polyvinylidene difluoride membrane. After incubation with 5% nonfat milk in TBST (Tris-buffered saline with 0.1% Tween 20 [Bio-Rad Laboratories, 170–6531]) for 1 h, membranes were incubated with various primary antibodies overnight at 4°C. Membranes were washed 3 times with TBST for 10 min followed by probing with secondary antibody for 1 h at room temperature. Blots were washed 3 times for 10 min with TBST and developed by using the Azure Biosystem C600 and band intensity was analyzed by Image Studio Lite (LiCor 5.2.5) according to the manufacturer<apos;>s manual.

### Generation of SIGMAR1/Sig1R-knockout Neuro2A cell line

Two of the pSpCas9 BB-2A-Puro (PX459) plasmids containing CRISPR guide RNA (gRNA) sequence targeting the mouse SIGMAR1/Sigma-1 receptor were obtained from GenScript (SC1948-459). The gRNA sequences, 5’-GGCCCCGGGCATAGGCCCGA-3’ and 5’-CGCTAGAATGCCGTGGGCCG-3’ were used (Table 1). Two plasmids were mixed in equal amount and transfected into Neuro2A cells. At 48 h after transfection, cells were treated with 2 µg/ml puromycin for 7 days. Cells were trypsinized and resuspended to a density of 8–10 cells/ml and 100 µl each of the cell suspension was transferred to a well of a 96-well plate. Expanded cells were collected and cell lysates were analyzed for the SIGMAR1/Sigma-1 receptor protein expression by western blot by using the Santa Cruz Biotechnology B5 anti-SIGMAR1/Sigma-1 receptor antibody (Table 1).

### Immunoprecipitation

Cell lysates from NSC34 cells were harvested in 0.3 ml of IP lysis buffer (50 mM NaCl, 0.5% Nonidet P-40 [Tergitol solution; SigmaAldrich, NP40S], 10 mM Tris-HCl, pH 8.0, and 1× protease inhibitor) for 30 min. Protein amounts were measured (Pierce bicinchoninic acid protein assay kit; Thermo Fisher Scientific, 23,235) after centrifugation (15,871xg for 10 min at 4°C). Protein lysate (200 μg or 450 μg) were mixed with specific antibody (2 μg) or control IgG was added into the lysates and rotated for 2 h at 4°C. Subsequently, the lysate containing antibody was added into protein-A/G agarose beads (50 μl; Santa Cruz Biotechnology, sc-2003) at a total volume of 1000 μl and rotated overnight at 4°C. The beads were washed three times with IP lysis buffer containing protease inhibitors for 5 min at 4°C with each wash accompanied by centrifugation at 9391 x g or 1 min at 4°C to remove the supernatant. After the 3^rd^ wash, bound proteins were eluted with 50 μl SDS 2X sample buffer containing 1% 2-ME and heated at 95°C for 10 min. The resulting proteins were immediately separated by using SDS/PAGE and immunoblotted with primary antibody overnight at 4°C. Membranes were washed 3 times for 10 min followed by probing with specific secondary antibody as described above. Blots were washed 3 times for 10 min with TBST and developed by using the Azure Biosystem C600. The band intensity was analyzed by Image Studio Lite (LiCor 5.2) according to the manufacturer<apos;>s manual.

### Protein degradation assay

Cycloheximide (100 µg/mL; Sigma-Aldrich, 01810) was added to the 80% confluent GFP-(G_4_C_2_)_31_-expressing NSC34 cells (previously transfected with HA or HA-SIGMAR1/Sigma-1 receptor) to inhibit *de novo* protein synthesis. Cells were harvested at different time points and lysed with modified RIPA lysis buffer as described in the western blot section. The lysate was examined by western-blot analysis by incubating overnight at 4°C with primary antibodies of target genes in TBST. After incubation with secondary antibodies (Jackson ImmunoResearch Laboratories, 115–001-008, 115–035-164, 115–035-174, 111–035-046), blots were imaged by Azure Biosystem C600 and band intensity was analyzed by Image Studio Lite (LiCor 5.2) according to the manufacturer<apos;>s manual.

### Nuclear-cytoplasmic fractionation

NSC34 cells were grown to 70% confluency and transiently transfected with indicated vectors including pEGFP-N3.31-(C_4_G_2_)_31_ (self-constructed by M. Cozzolino), pCMV3-HA (Sino Biological, CV017), pCMV3-HA-SIGMAR1/Sigma-1 receptor (Sino Biological, MG57873-NY), pCMV6-MYC/DDK (Origene Technologie, Inc., PS100001) or pCMV6-POM121-MYC/DDK (Origene Technologies, Inc., MR211792). Twenty-four hours after transfection, cells were harvested for subcellular fractionation using the Subcellular Protein Fraction Kit for Cultured Cells (Thermo Fisher Scientific, 87,790). The procedure is briefly described per manufacturer<apos;>s instructions as follows. Transfected cells were rinsed once with PBS and vortex-lysed with cytoplasmic extraction buffer that was supplemented with protease inhibitor cocktail for 20s. The cytoplasmic fraction (supernatant) was collected by centrifugation at 500 × g for 5 min at 4°C. The pellets were then incubated with membrane extraction buffer at 4°C for 10 min and the membrane fraction (supernatant) was prepared by centrifugation at 3000 × g for 5 min at 4°C. For the isolation of the nuclear extract, the resultant pellets were subsequently incubated with nuclear extraction buffer at 4°C for 30 min and the soluble nuclear extract (supernatant) were collected by centrifugation at 5000 × g for 5 min at 4°C.

### Fluorescence in situ hybridization for (G4C2) repeats

Briefly, EGFP or EGFP-(G_4_C_2_)_31_-expressing NSC34 cells were grown on poly-L-lysine-coated glass coverslips. After 24 h, the glass coverslips were washed in PBS and fixed with 4% paraformaldehyde in PBS for 10 min. Further, cells were washed twice with 70% ethanol and stored in 70% ethanol at 4°C for 30 mins. Cells were rehydrated in PBS containing 5 mM MgCl_2_ for 30 min and then pre-hybridized in 35% formamide, 10 mM sodium phosphate pH 7.0, and 2x SSC (SSC Buffer 20x concentrate; SigmaAldrich, S6639) for 30 min at room temperature. For the probe hybridization, cells were incubated with 250 ng/ml of Cy3-labeled (C_4_G_2_)_4_ (Integrated DNA Technologies, Custom-made) in 30% formamide, 10% dextran sulphate, 2x SSC, 0.2% BSA, 10 mM sodium phosphate pH 7.0, and 0.5 mg/ml *E. coli* tRNA (from baker<apos;>s yeast; Roche Diagnostics, 10,109,495,001) at 37°C for overnight. After hybridization, cells were washed twice with 35% formamide, 10 mM sodium phosphate, pH 7.0, and 2x SSC for 30 min each at 37°C; twice with 2x SSC, 0.1% Triton X-100, 15 min each at room temperature; and twice with 0.2x SSC, 0.1% Triton X-100, 15 min each at room temperature. After washing, coverslips were visualized by confocal microscopy.

### SIGMAR1/Sigma-1 receptor agonist assay determining the dissociation of SIGMAR1/Sigma-1 receptor from HSPA5/BiP: A modified method utilizing transiently transfected and not permanently transfected SIGMAR1/Sigma-1 receptor-YFP cells.

Our original method utilizes cells permanently transfected with SIGMAR1/Sigma-1 receptor-YFP to detect its interaction with HSPA5 [28]. However, it is not always easy to obtain a cell line permanently expressing transfected gene. Instead, we found in this study that simply by utilizing transiently transfected cells (20–24 h) we can successfully detect the association between SIGMAR1/Sigma-1 receptor-YFP and HSPA5 with very low background as shown in Figure 9(a). The rest of the procedures are essentially the same as described in the method for immunoprecipitation (see above). However, in this modified method it is essential to use horseradish peroxidase-conjugated secondary antibody, and not fluorescent secondary antibody, in order to largely enhance the detection sensitivity with low background signals.

### SIGMAR1/Sigma-1 receptor oligomerization assay using non-denaturing gels

NSC34 cells pretreated with pridopidine (Prilenia Therapeutics inhouse product; Pridopidine, Batch 0000011658) for 1 h were harvested by the lysate buffer per the instruction of the NativePAGE^TM^ Sample Prep Kit (NOVEX, BN2008). Protein amounts were measured (Pierce bicinchoninic acid protein assay kit; Thermo Fisher Scientific, 23,235) after centrifugation (20,000 x g for 20 min at 4°C). Protein lysates (25 μg) were loaded in the NativePAGE^TM^ Novex® 4–16% Bis-Tris gel (NOVEX, BN1002BOX) for electrophoresis. The resultant gels were transferred (300 mA for 2 h) to PVDF membrane followed by immunoblotting with SIGMAR1/Sigma-1 receptor antibody (B5, Table 1) overnight at 4°C. Membranes were washed 3 times for 10 min followed by probing with specific secondary antibody. Blots were washed 3 times for 10 min with TBST and developed by using the Azure Biosystem C600 and band intensity was analyzed by Image Studio Lite (LiCor 5.2) according to the manufacturer<apos;>s manual. Note: under the conditions employed in this assay per manufacturer<apos;>s instructions, molecular weight markers could not transfer from gel into the PVDF membrane. Therefore, after the native gel electrophoresis, the lane with markers was cut and stained directly with Coomassie Brilliant Blue. The resultant stains of those markers were used to indicate the molecular size of various SIGMAR1/Sigma-1 receptor oligomers.

### GST-SIGMAR1/Sigma-1 receptor protein purification

pGEX-6p3-human SIGMAR1/Sigma-1 receptor construct was self-made and obtained as shown in our previous study [32]. pGEX-6p3-human SIGMAR1/Sigma-1 receptor vectors (inhouse production, T-P Su Lab; see [31]) were transformed into BL21 (DE3) *E. coli* (New England BioLabs Inc., C2527H) and incubated in LB agar plate containing 100 µg/ml ampicillin at 37°C overnight. After 16 h, selected colonies were cultured in 3 ml of LB broth containing 100 µg/ml ampicillin at 37°C overnight. After 16 h, growth bacterial solution was separated into two different tubes in 3 ml of LB broth containing 100 µg/mL ampicillin and grown until A_600nm_ reaches 0.6 followed by treatment with or without 1 mM IPTG (SigmaAldrich, I6758) at 25°C overnight. To check the success of induction, 1 ml of bacterial solution was centrifuged at 3381 x g for 2 min, pellets were resuspended in 2× sample buffer containing 2-ME and denatured at 95°C for 10 min. Twelve percent SDS-page was conducted to check the success of induction. In the large-scale preparation, bacterial colonies of high induction were incubated in 400 ml of LB broth containing 100 µg/ml ampicillin and 1 mM IPTG at 25°C overnight. The lysates were harvested in 20 mL PBS (pH 7.4) containing 0.1% Triton X-100 and then sonicated for 30 min. After sonication, lysates were incubated at 4°C overnight. For affinity purification, the glutathione-Sepharose resins were poured into a column and washed with 3 × 10 ml of PBS. Twenty ml of lysates were slowly flowed into the column which was then eluted with 5 ml of elution buffer (10 mM glutathione in reduced form [SigmaAldrich, G4251], 50 mM Tris-HCl, pH 8.0) to obtain purified proteins. The eluants were then placed into a regenerated cellulose tubular membrane and dialyzed against S100 buffer (25 mM HEPES, 20% glycerol [SigmaAldrich, G5516], 100 mM KCl, 0.2 mM EDTA, 1 mM DTT) at 4°C overnight. Protein amounts were measured by Pierce bicinchoninic acid protein assay kit (Thermo Fisher Scientific, 23,235). Purified proteins in aliquots were stored at −80°C.

### Chaperone activity assay measuring the aggregation of citrate synthase using purified proteins

Briefly, 1.1 mM of CS (citrate synthase; Sigma-Aldrich, C-3260) in 100 ml of 50 mM HEPES-NaOH buffer (pH 7.5) was heated at 45°C in the presence of GST or GST-SIGMAR1/Sigma-1 receptor (1 mM) with or without pridopidine (0.2 mM) or BD1063 (0.2 mM). The scattering of light due to the CS aggregation was measured at 320 nm for 70–90 min by using SpectraMax M2 (Molecular Devices, San Jose, CA) detection.

### GFP-TFEB immunofluorescence assay

NSC34 cells were transfected with GFP-TFEB vector (Origene, MR223018L4) in 10% FBS DMEM medium overnight. Afterwards, the transfected cells were switched to serum-free medium for 1 h and then fixed with 4% paraformaldehyde at room temperature for 20 min. Cells were then washed three times with PBS and mounted with Prolong gold antifade mountant (Invitrogen, P36961) with DAPI (Cell Signaling Technology, 4083S). Images of cells were captured by confocal microscopy (Perkin- Elmer Modular laser system 2.0 with Nikon Eclipse TE2000E microscope and Volocity version 6.3 software). The quantification of nuclear GFP-TFEB intensity was performed by using NIH ImageJ. (version 1.51b). Pridopidine was given 1 h before transfection and remained in the medium throughout the experiment. For the transfection of (G4C2)_31_ and GFP-TFEB plasmids when examining the pridopidine effect, 3 µg of each plasmid was used. For the transfection of 3 plasmids, 2.5 µg per plasmid was used.

### Cell viability assay examining the toxic effect, if any, of (G4C2)RNA repeats and blockade of its toxicity by pridopidine

NSC34 cells were grown in 96-well transparent plate and treated with or without 1 µM Pridopidine for 1 h. Subsequently, cells were transfected with (G4C2)-RNA 106 or 288 repeats for 24 h. Note: Both (G4C2)_106_ and (G4C2)_288_ are RNA only constructs. After 24 h, cells were treated 100 µM H_2_O_2_ for 3 h. The media were removed from experimental cells and replaced with reagent from Cell Counting Kit-8 (Abcam, ab228554) per manufacturer<apos;>s instructions for 3 h. The absorbance increase indicating live cells was then measured spectrophotometrically at 460 nm using an ELISA plate reader.

### Statistics and reproducibility

For all experiments subjected to statistical analyses, data were collected from at least three independent experiments and were compared for statistical significance by using Prism (version 8.2). No samples were pre-allocated to specific groups to maintain randomization. Data were collected from experiments performed in replicates and were expressed as means ± SEM. Unpaired Student<apos;>s t-test, one-way ANOVA, or two-way ANOVA followed by appropriate post hoc test were used to test the statistically significant. For comparisons between non-linear regression curves (i.e., Figure 4(b-f)), the second order polynomial (quadratic) model was first used for the curve fit. Next, the “extra sum-of-squares F-test” was used to test if the best-fit curve of a group is the same as the global (shared) fitting curve. A *p*-value *≤* 0.05 was considered statistically significant.

## Supplementary Material

Supplemental Material

## Data Availability

All materials requests and correspondence are to be addressed to the corresponding author, Dr. Tsung-Ping Su. All original data are available upon reasonable request.

## Subject classification

Neuroscience
