# Supplementary material for: Nucleoporin POM121 signals TFEB-mediated autophagy via activation of SIGMAR1/sigma-1 receptor chaperone by pridopidine
Source: Autophagy. 2022 May 4;19(1):126–51. doi: 10.1080/15548627.2022.2063003 (PMC9809944; doi:10.1080/15548627.2022.2063003)
Supplement: Supplemental Material [file KAUP_A_2063003_SM1024.docx]

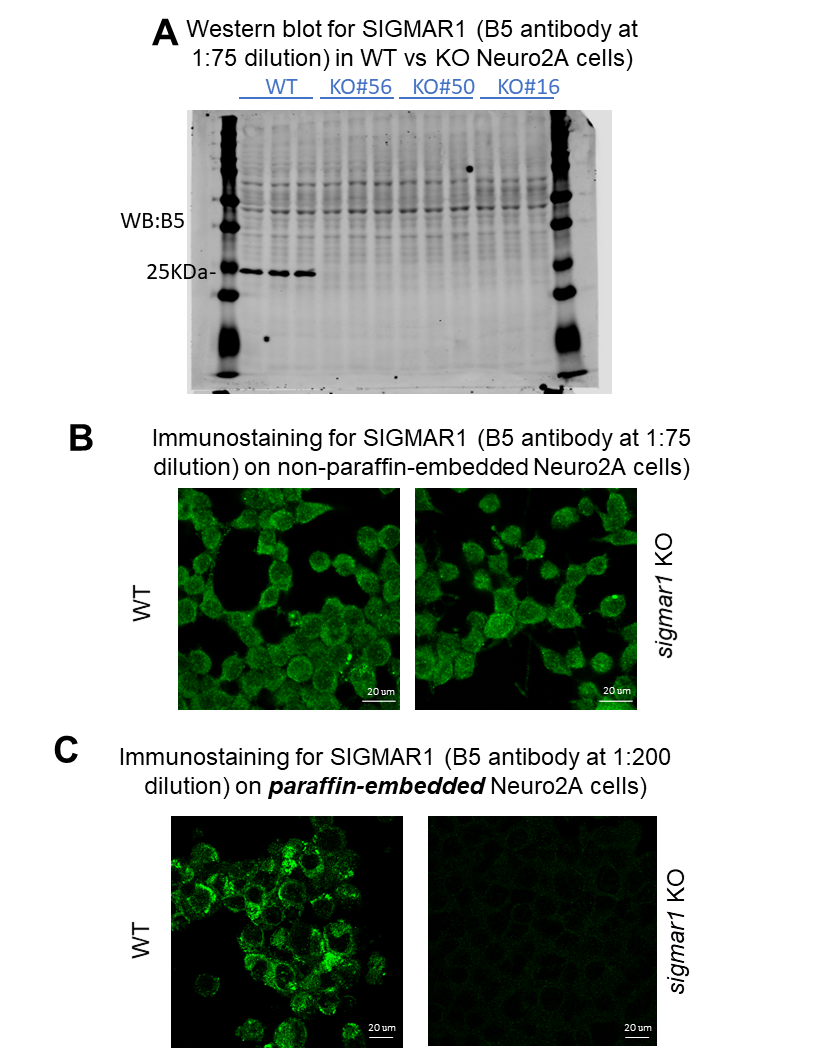


**Figure S1.** Validation of the specificity of the antibody against SIGMAR1/Sigma-1 receptor used in Figure 1. Santa Cruz Biotechnology anti-SIGMAR1 monoclonal antibody B5 (B5: sc137075) against SIGMAR1 was used in figure 1. The B5 antibody specificity against SIGMAR1 was examined in this figure by using wild type and *sigmar1* knockout Neuro2A cells which are readily available in the lab. (**A**) In Neuro2A cells with CRISPR-CAS9-based knockout (KO) of *Sigmar1*, no SIGMAR1 was detected by B5 in the three KO cell groups (right nine sample lanes) when compared to WT cells (left three sample lanes). (**B**) Regularly processed, non-paraffin-embedded, Neuro2A cells were used to check the specificity of B5. B5 failed to show specificity and recognized SIGMAR1 in both WT and *sigmar1* KO cells. (**C**) In paraffin-embedded Neuro2A cells (see details on Methods), B5-positive signals were clearly seen in WT cells but not in *sigmar1* KO cells.


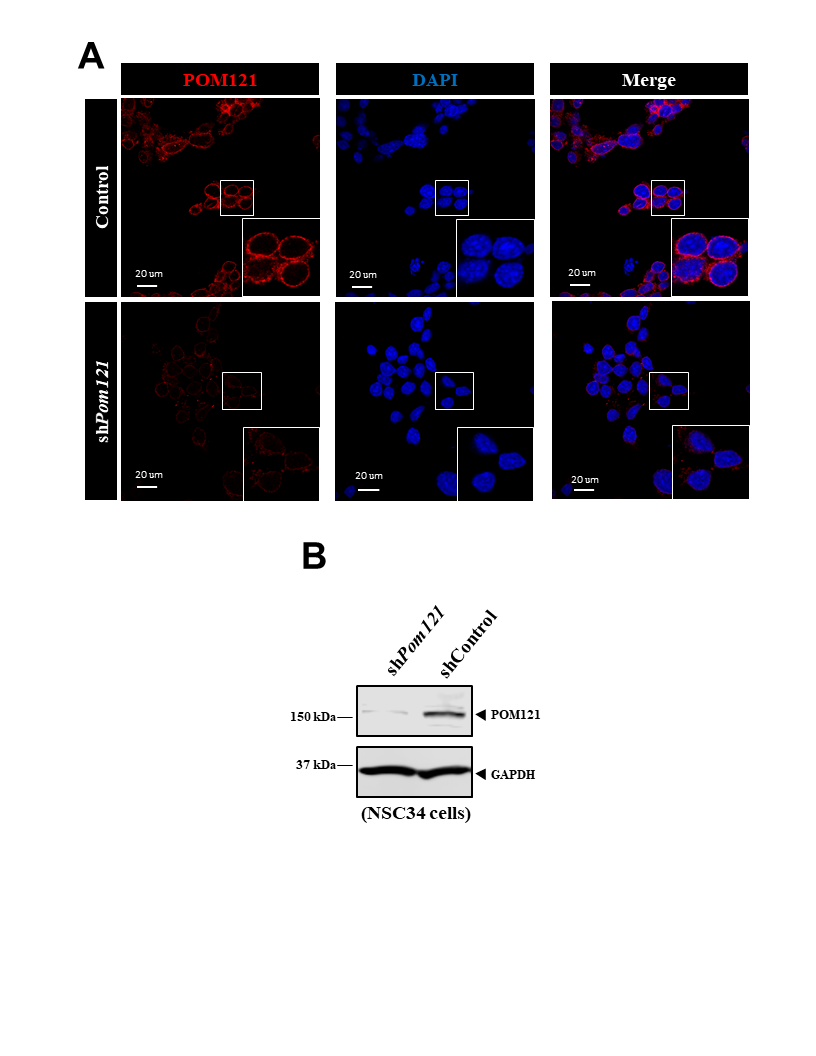


**Figure S2.** Validation of the specificity of the antibody against POM121 used in Figure 1. The antibody against POM121 that was used in figure 1, as well as in this figure, was from Novus Biologicals (NB, 2-19890). (**A**) Knockdown of *Pom121* by using shRNA (Santa Cruz Biotechnology; sc-152388-SH; Lot #B2714) largely reduced the POM121 immunofluorescence. Note some nonspecific signals in the knockdown cells. (**B**) Western blot also showed minor nonspecific proteins. Nevertheless, majority of immunofluorescence were from POM121.

**
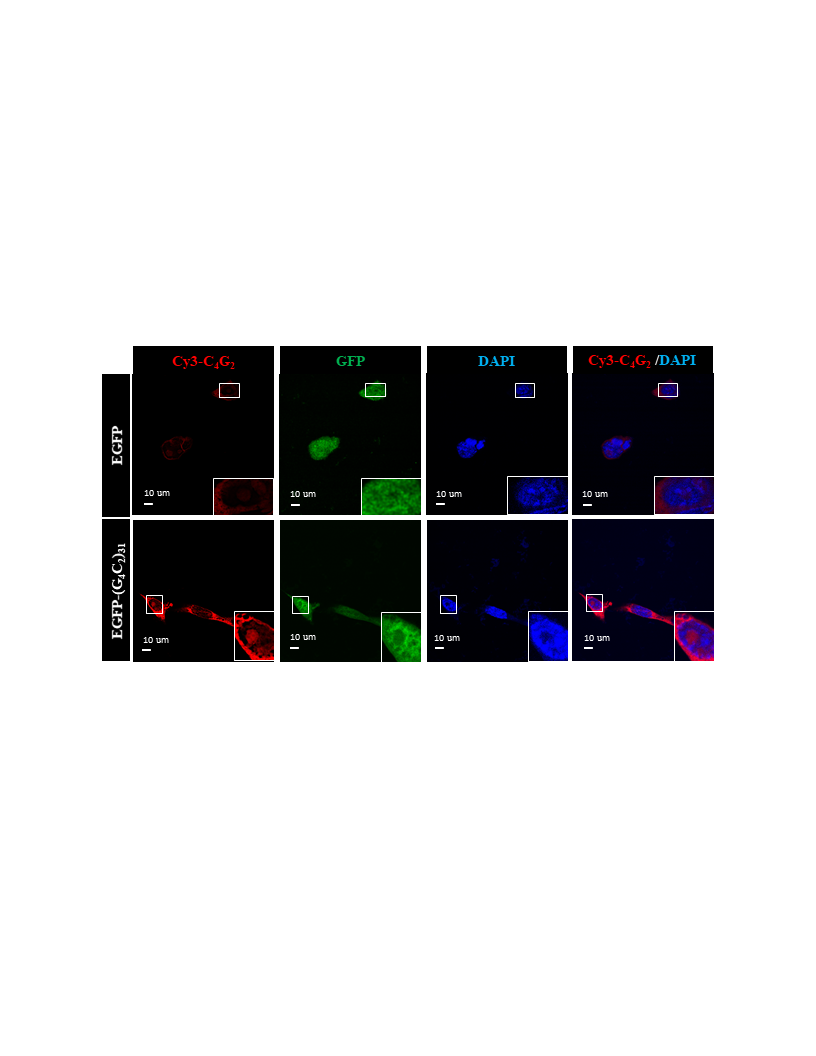
**

**Figure S3.** Successful transfection of EGFP-(G_4_C_2_)_31_ validated by fluorescent in situ hybridization assay. NSC34 cells were transfected with EGFP or EGFP-(G_4_C_2_)_31_ vectors. Cells were fixed and hybridized with Cy3-(C_4_G_2_)_4_ (Red) in the RNA fluorescent in situ hybridization assay.


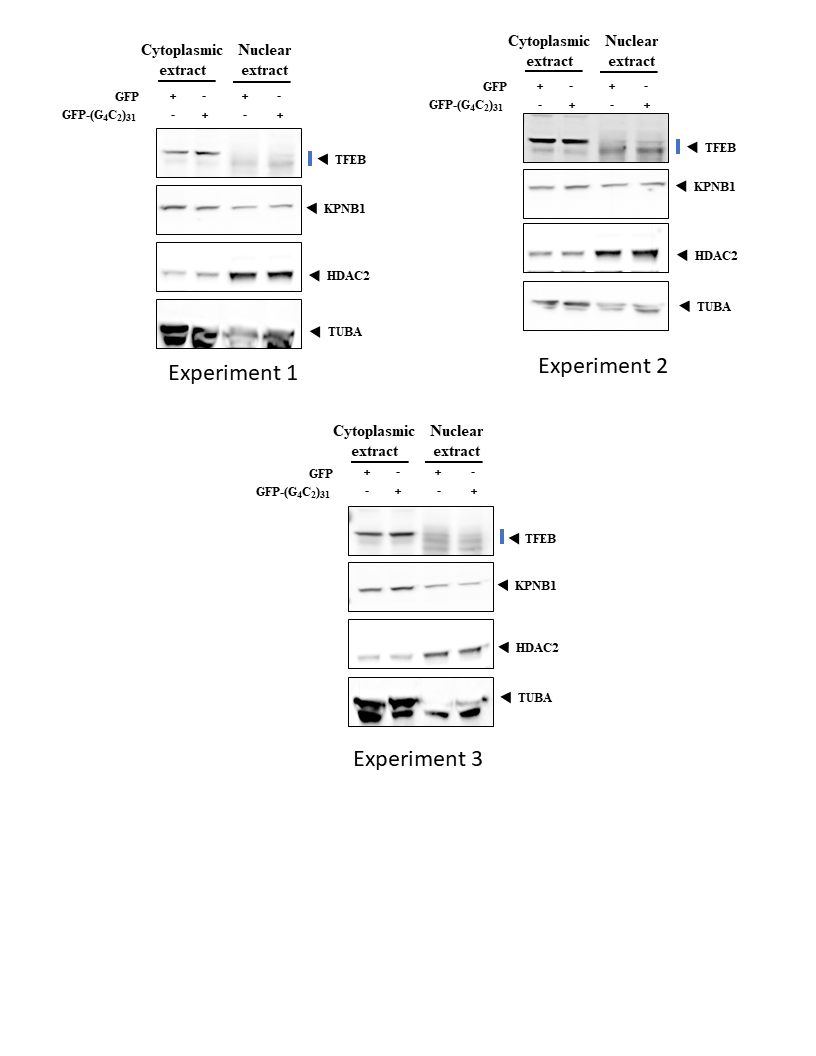


**Figure S4.** Three repetitions of the effect of (G4C2)_31_ on TFEB distribution (i.e., Fig. 6A).


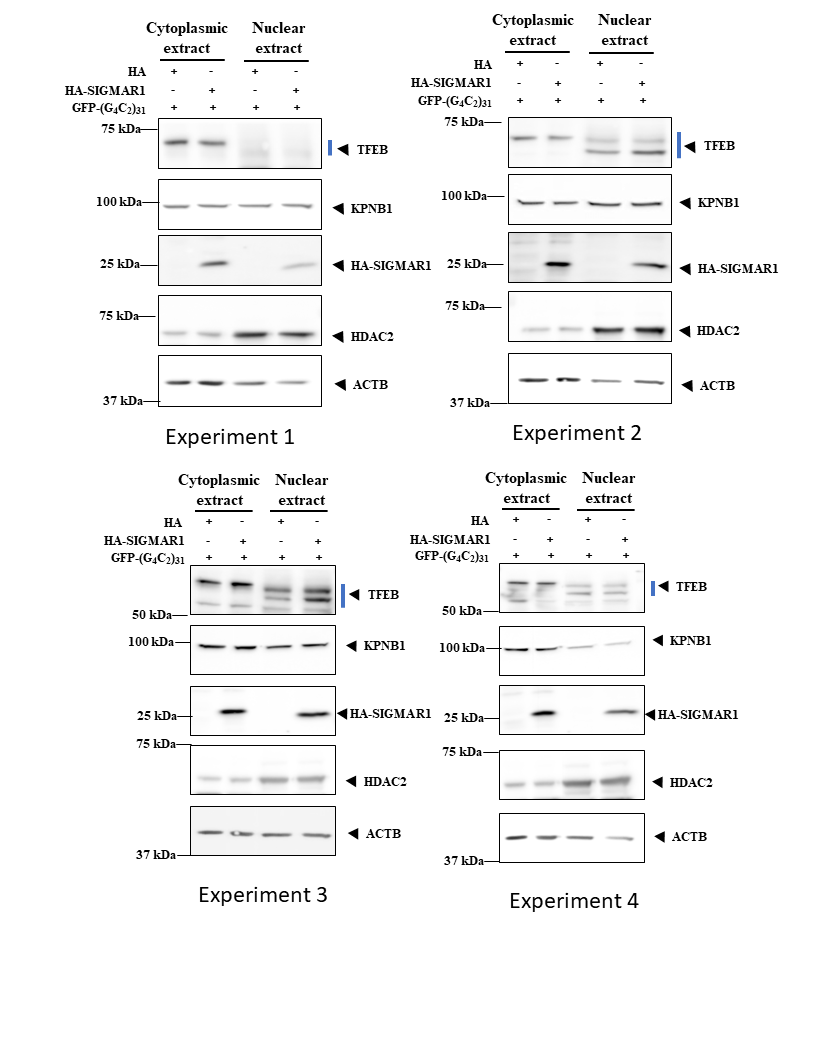


**Figure S5.** Four repetitions of the effect of SIGMAR1/Sigma-1 receptor overexpression on (G4C2)31-induced alternation of TFEB distribution (i.e., Fig. 6C).


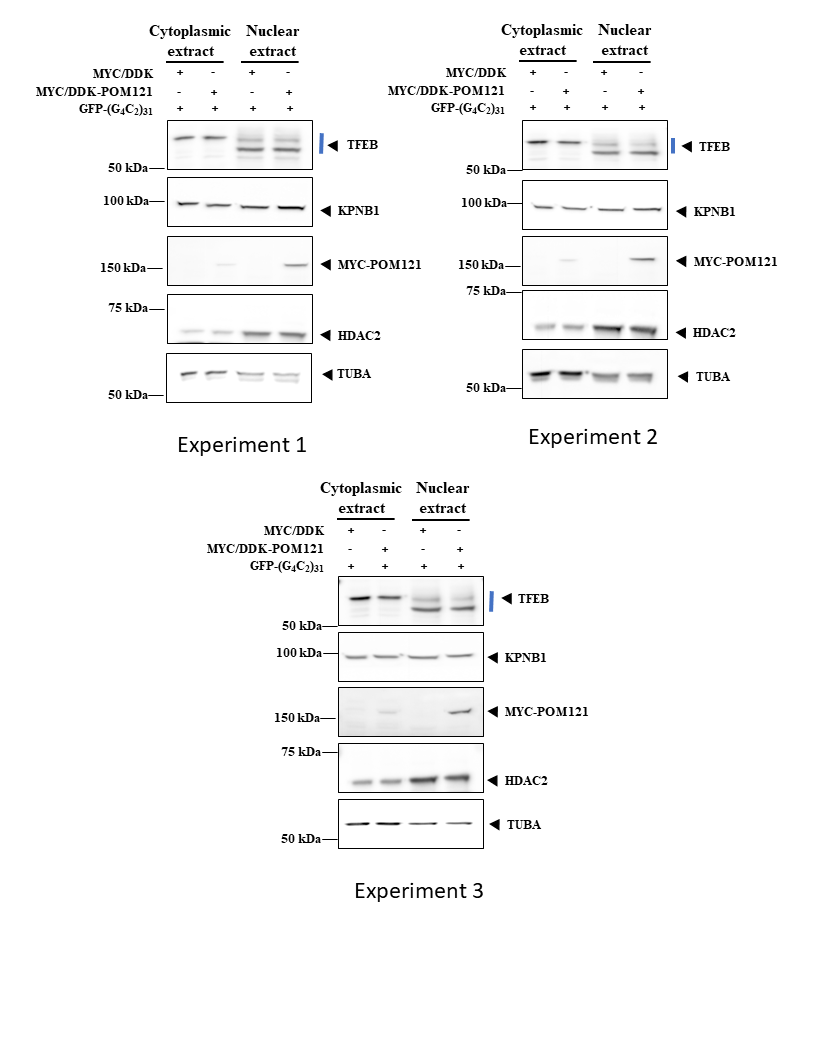


**Figure S6.** Three repetitions of the effect of POM121 overexpression on (G4C2)31-induced alternation of TFEB distribution (i.e., Fig. 6E).


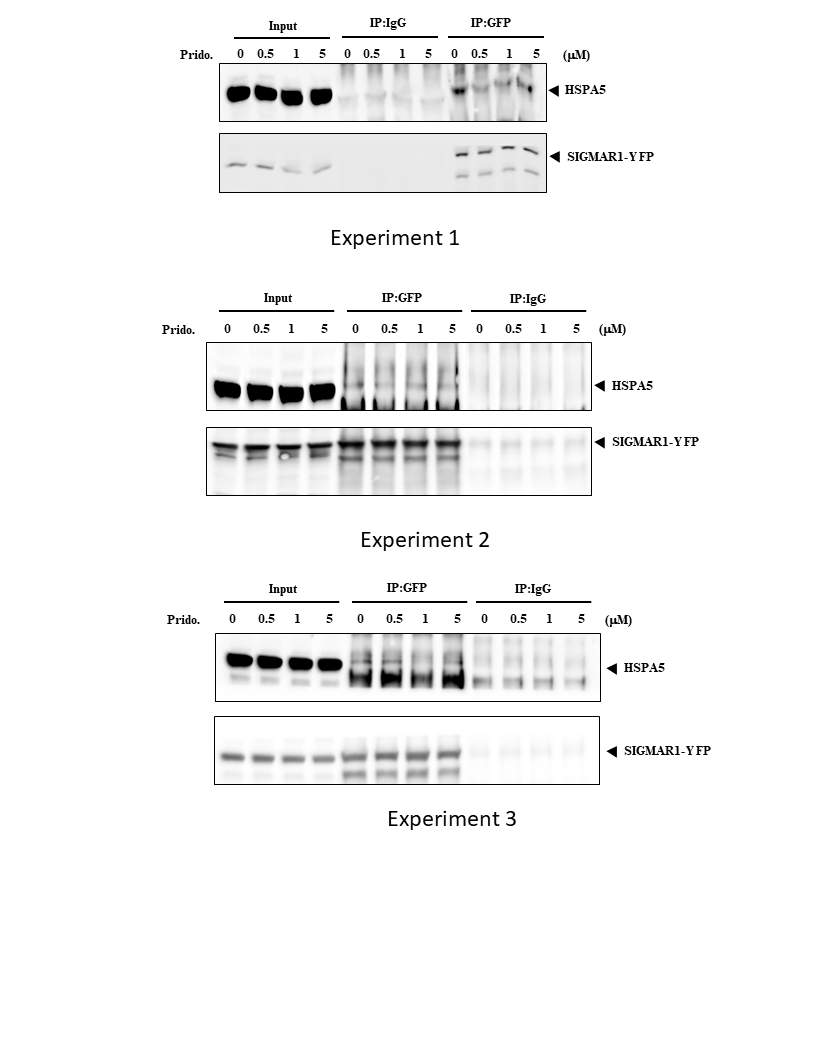


**Figure S7.** Three repetitions of SIGMAR1/Sigma-1 receptor-HSPA5/BiP dissociation assay in the presence of pridopidine (i.e., Fig. 9A).


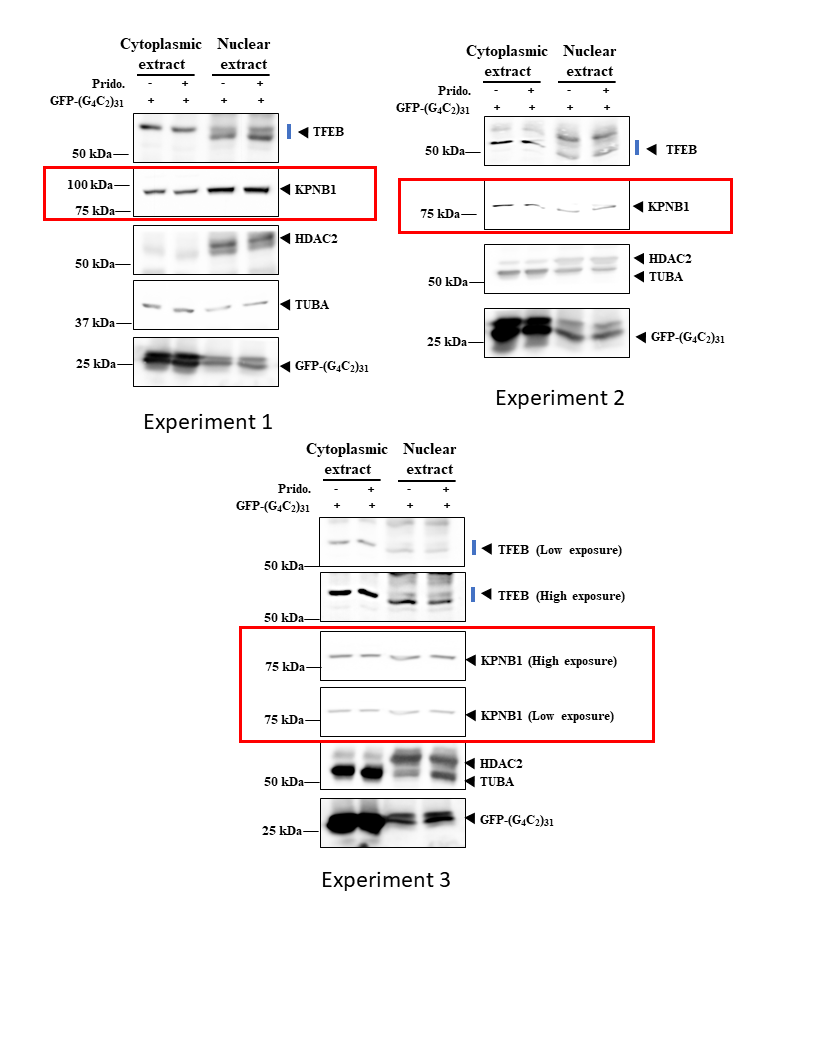


**Figure S8.** Three repetitions of the effect of pridopidine on TFEB distribution imposed by (G4C2)31 (i.e., Fig. 11D).


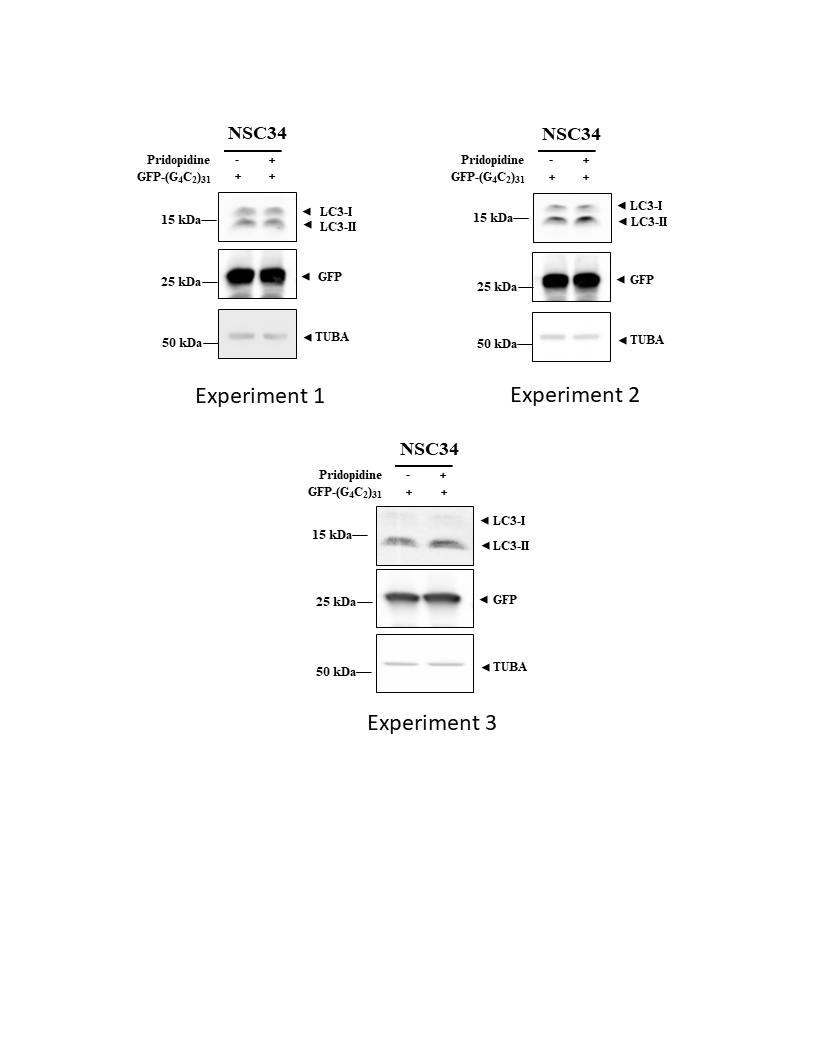


**Figure S9.** Three repetitions of the effect of pridopidine on LC3-I and LC3-II (i.e., Fig. 11G).
